# Supplementary material for: IF1 is a cold-regulated switch of ATP synthase hydrolytic activity to support thermogenesis in brown fat
Source: EMBO J. 2024 Sep 16;43(21):6. doi: 10.1038/s44318-024-00215-0 (PMC11535227; doi:10.1038/s44318-024-00215-0)
Supplement: Supplementary file 1 — Appendix [file 44318_2024_215_MOESM1_ESM.pdf]

Appendix

IF1 is a cold-regulated switch of ATP synthase hydrolytic activity to support thermogenesis in brown fat

Henver S. Brunetta, Anna S. Jung, Fernando Valdivieso-Rivera, Stepheny C. C. Zani, Joel Guerra, Vanessa Furino, Annelise Francisco, Marcelo Berçot, Pedro M. Moraes-Vieira, Susanne Keipert, Martin Jastroch, Laurent O. Martinez, Carlos H. Sponton, Roger F. Castilho, Marcelo A. Mori, Alexander Bartelt

Table of contents:

Appendix Table S1 Page 2
Appendix Table S2. Page 3

Appendix Table S1 – primer sequences.

Gene	Forward sequence	Reverse sequence
Tbp	AGA ACAATCCAGACTAGCAGCA	GGGAACTTCACATCACAGCTC
Atp5if1	GGTTCGGTGTCTGGGGTATG	ATCCATGCTATCCGACGAGT
Ucp1	AGGCTTCCAGTACCATTAGGT	CTGAGTGAGGCAAAGCTGATTT
Virus titration	CCCACTTGGCAGTACATCAA	GCCAAGTAGGAAAGTCCCAT

Appendix Table S2 – Antibodies for immunoblot.

Target antigen	Clonality	Vendor	Cat Number	Dilution
ATP5IF1	Polyclonal	Cell Signalling	8528	1:500
OXPHOS Cocktail	Monoclonal	Abcam	110413	1:1000
GAPDH	Monoclonal	Cell Signalling	5174	1:2000
p38MAPK	Polyclonal	Cell Signalling	9212	1:2000
p-p38MAPK	Polyclonal	Cell Signalling	9211	1:1000
Vinculin	Unknown	Cell Signalling	4650	1:5000
α-tubulin	Monoclonal	Sigma-Aldrich	6074	1:2000
β-tubulin	Polyclonal	Cell Signaling	2146	1:1000
