# Supplementary figures and images for: IF1 is a cold-regulated switch of ATP synthase hydrolytic activity to support thermogenesis in brown fat
Source: EMBO J. 2024 Sep 16;43(21):6. doi: 10.1038/s44318-024-00215-0 (PMC11535227; doi:10.1038/s44318-024-00215-0)

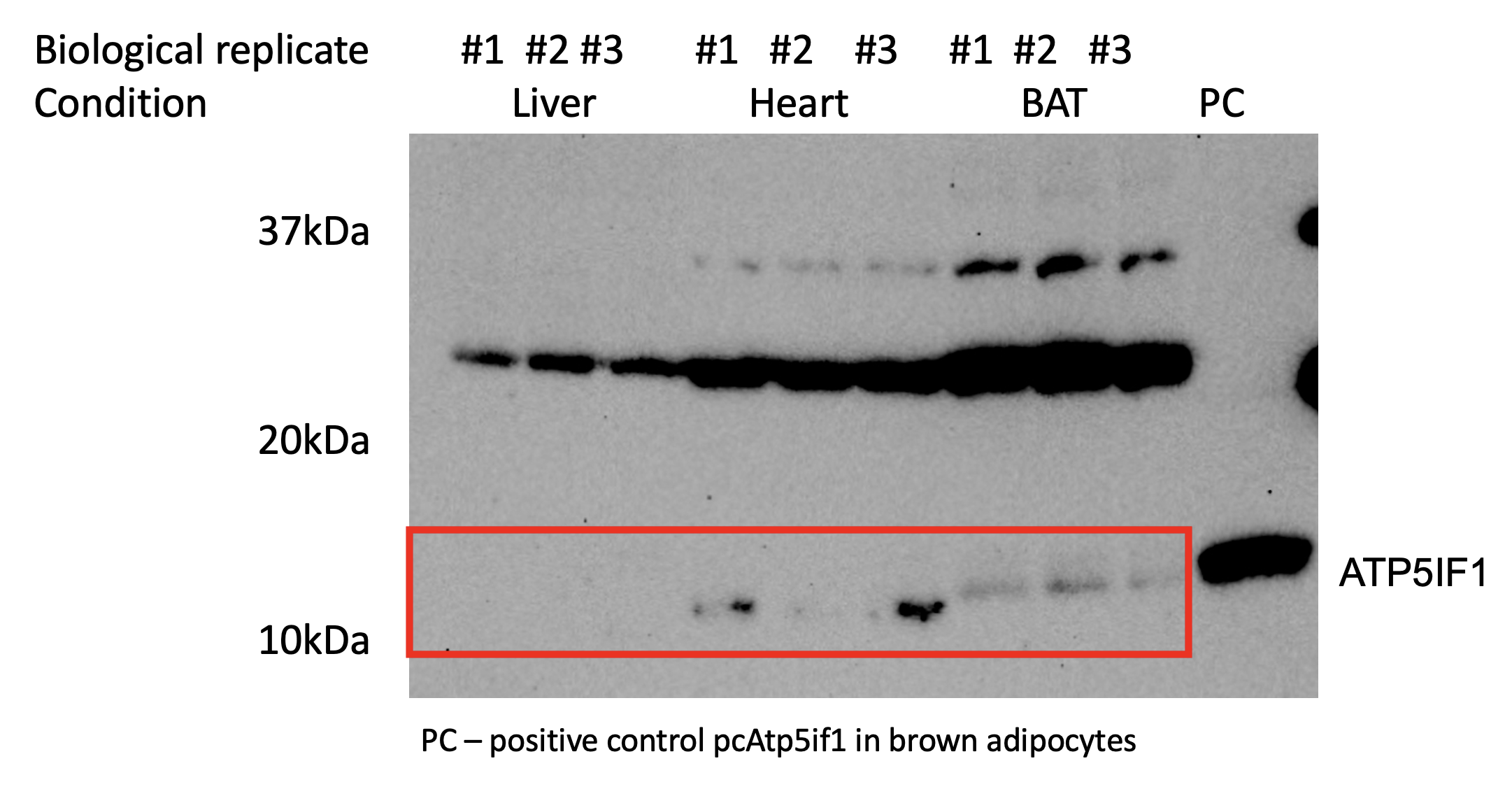

Supplement: Supplementary file 3 — Source data Fig. 1 [file 44318_2024_215_MOESM3_ESM.zip › Figure 1 38/1F/ATP5IF1 three tissues with positive control.png]

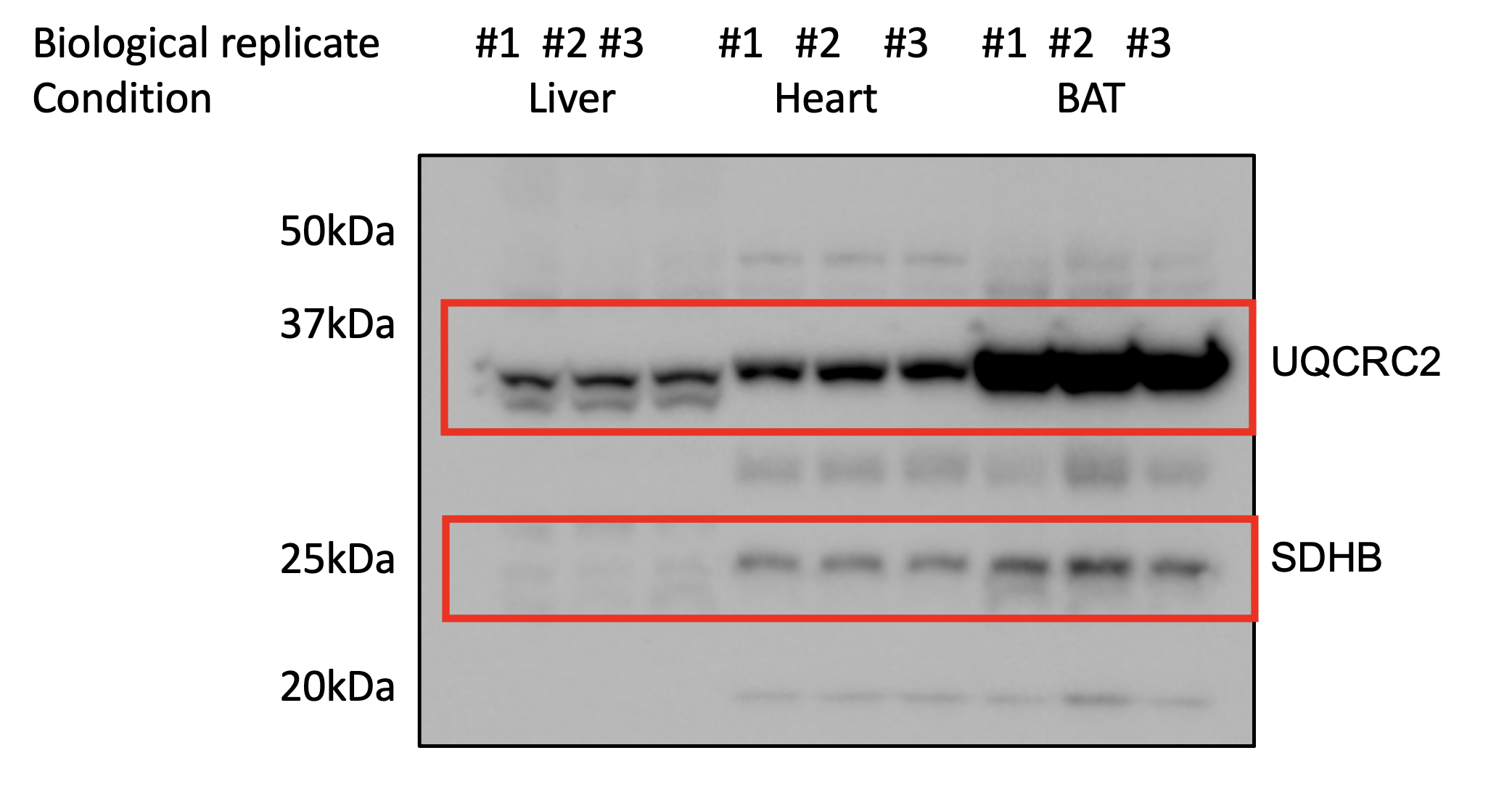

Supplement: Supplementary file 3 — Source data Fig. 1 [file 44318_2024_215_MOESM3_ESM.zip › Figure 1 38/1F/Complex III and II three tissues.png]

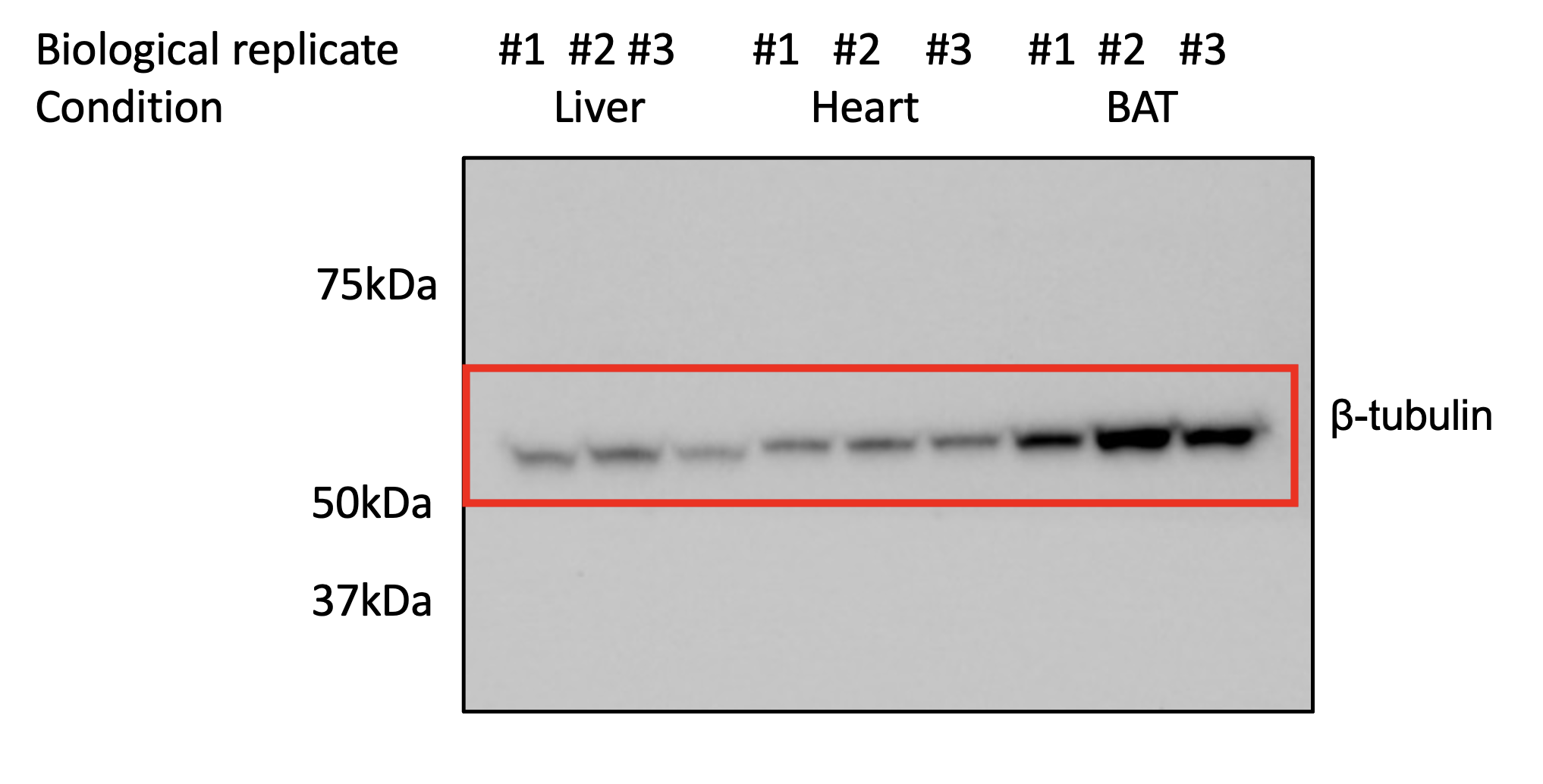

Supplement: Supplementary file 3 — Source data Fig. 1 [file 44318_2024_215_MOESM3_ESM.zip › Figure 1 38/1F/beta tubulin three tissues.png]

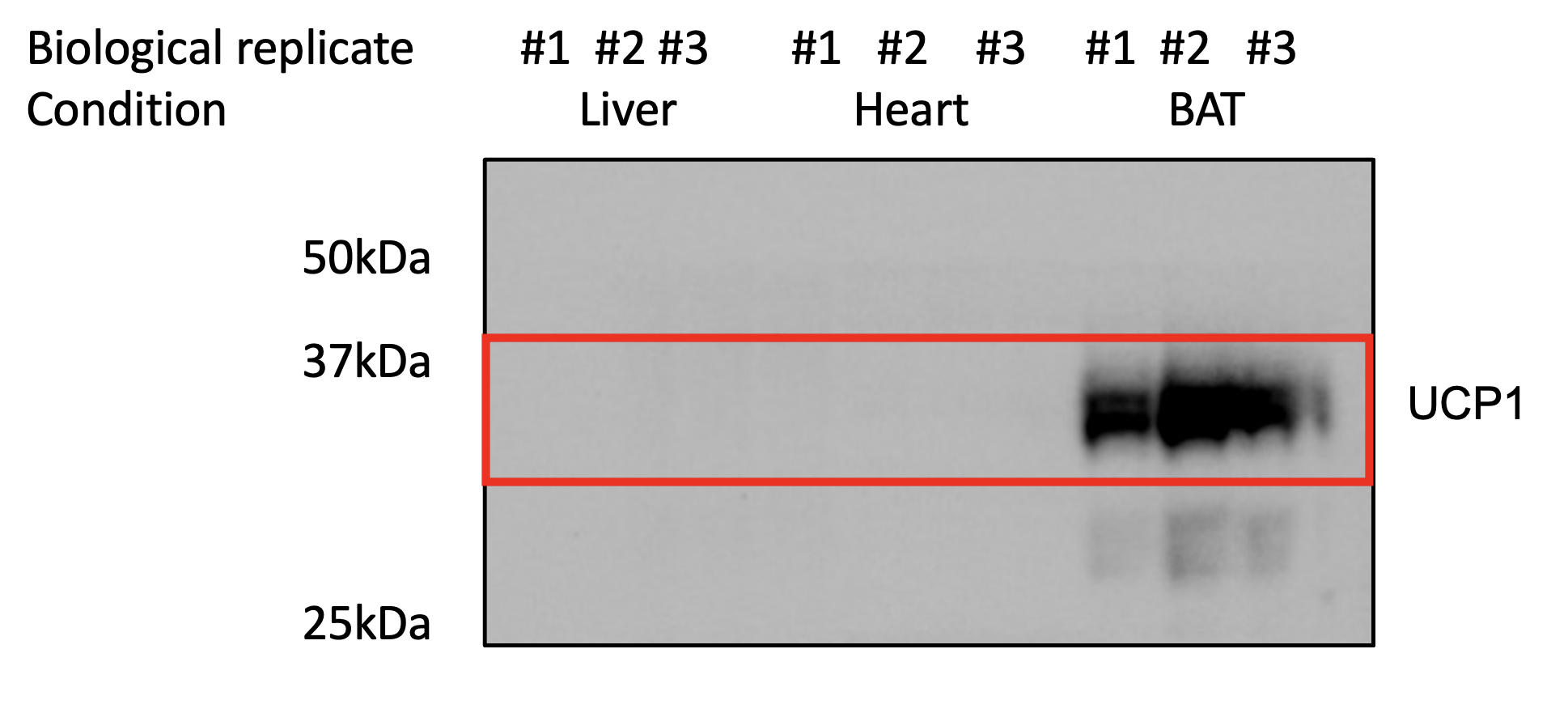

Supplement: Supplementary file 3 — Source data Fig. 1 [file 44318_2024_215_MOESM3_ESM.zip › Figure 1 38/1F/UCP1 three tissues.png]

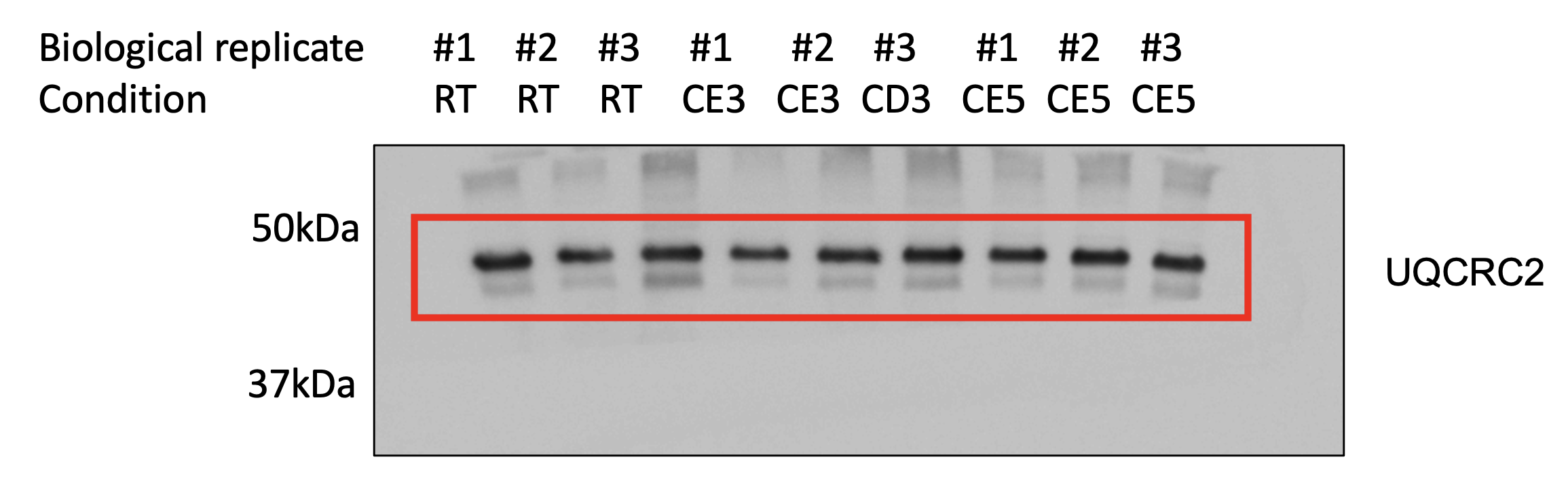

Supplement: Supplementary file 3 — Source data Fig. 1 [file 44318_2024_215_MOESM3_ESM.zip › Figure 1 38/1I/Complex III BAT cold exposure.png]

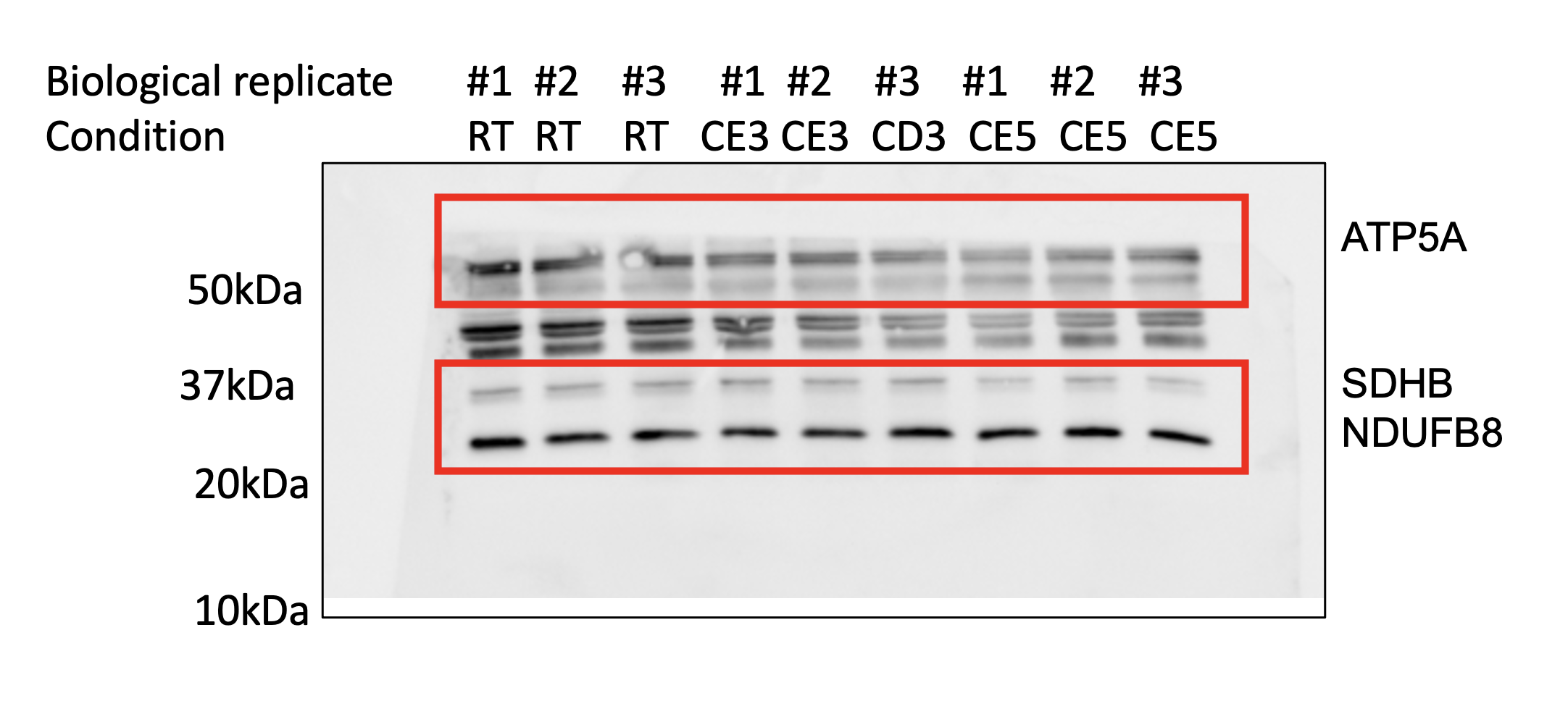

Supplement: Supplementary file 3 — Source data Fig. 1 [file 44318_2024_215_MOESM3_ESM.zip › Figure 1 38/1I/Complex V, II, and I - cold exposure BAT.png]

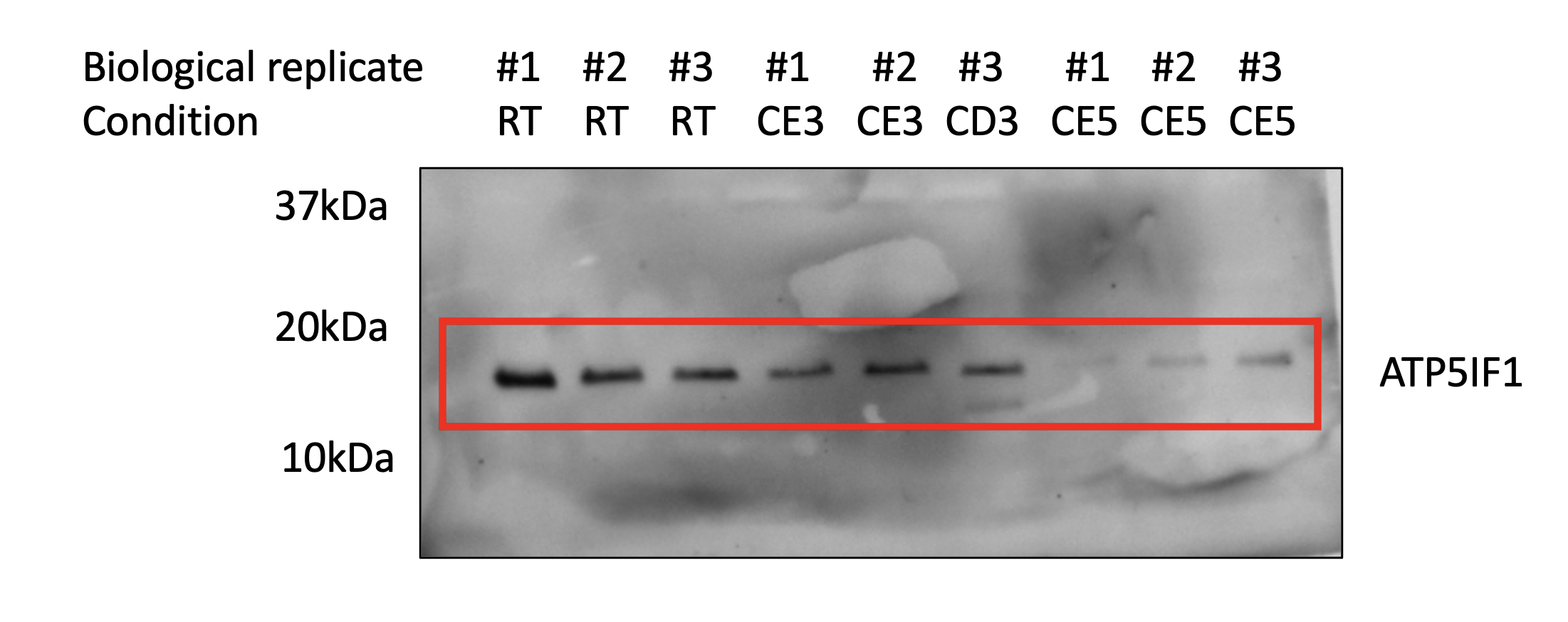

Supplement: Supplementary file 3 — Source data Fig. 1 [file 44318_2024_215_MOESM3_ESM.zip › Figure 1 38/1I/ATP5IF1 BAT cold exposure.png]

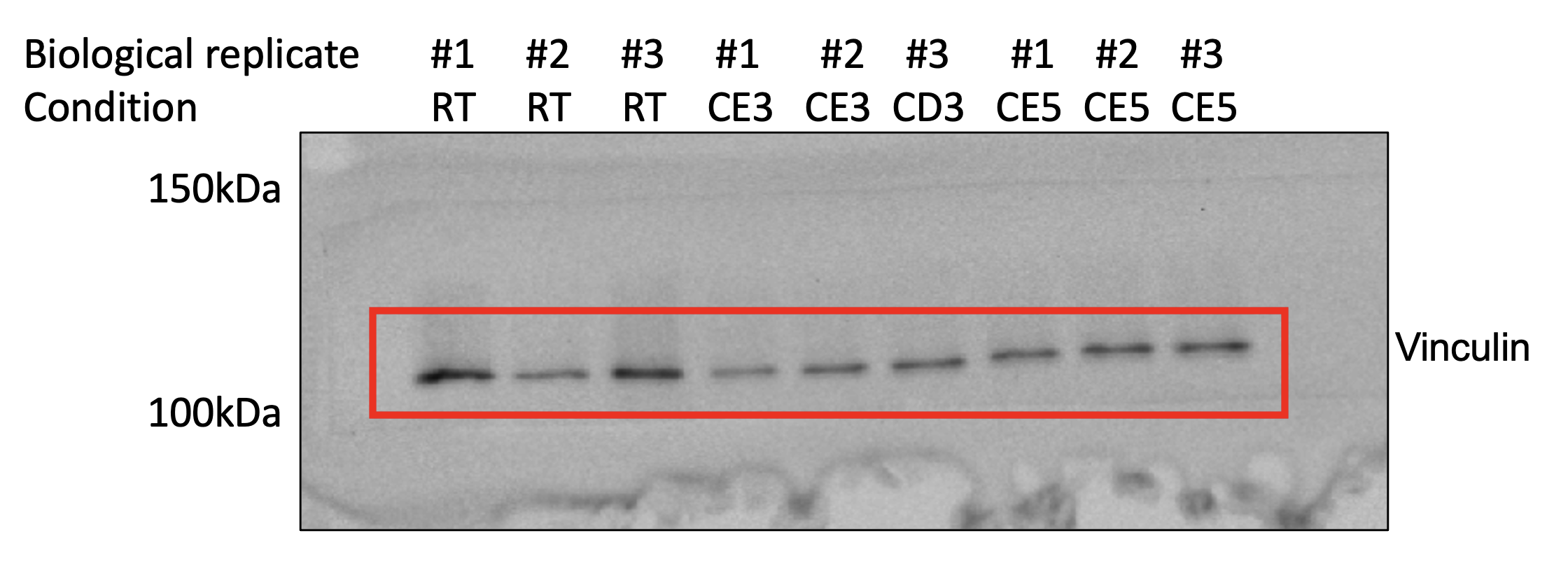

Supplement: Supplementary file 3 — Source data Fig. 1 [file 44318_2024_215_MOESM3_ESM.zip › Figure 1 38/1I/Vinculin BAT cold exposure.png]

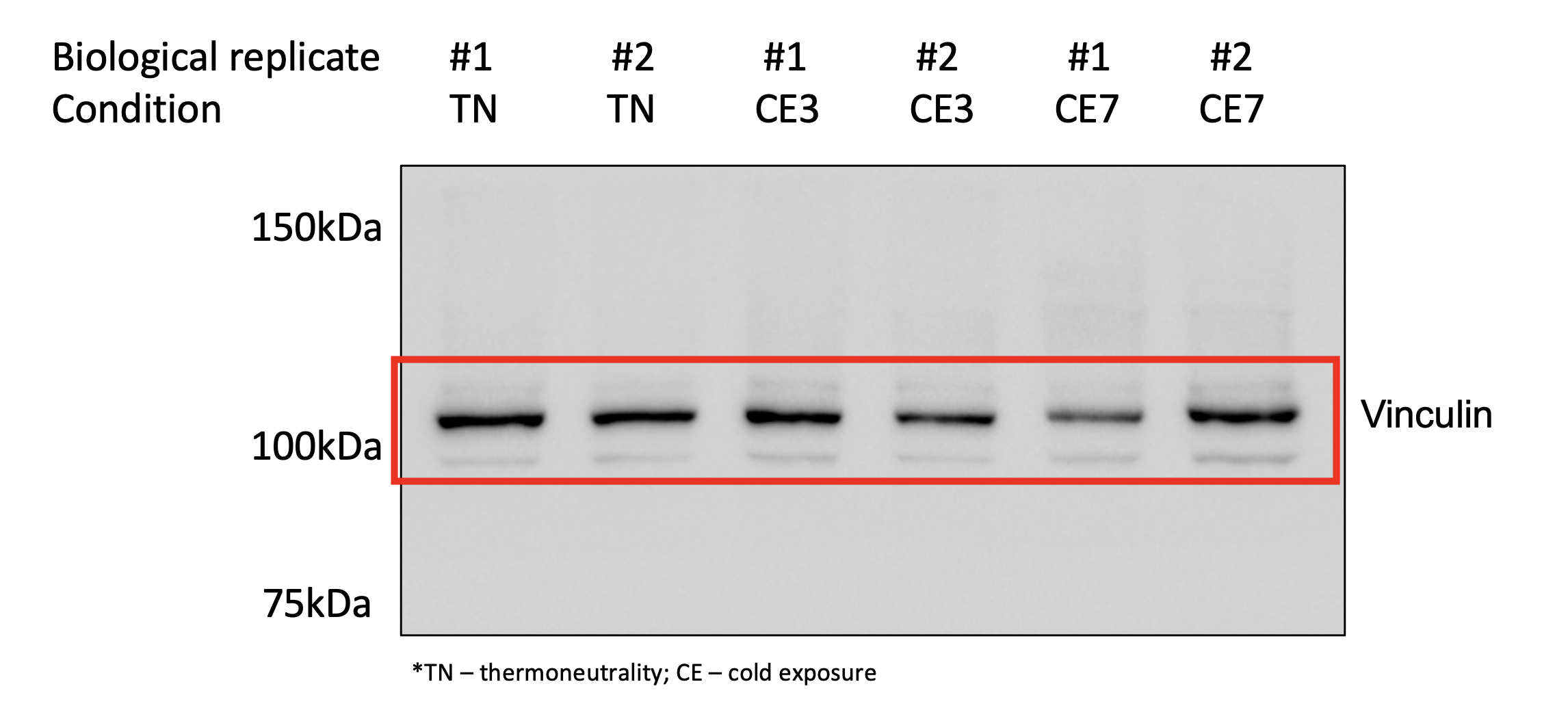

Supplement: Supplementary file 3 — Source data Fig. 1 [file 44318_2024_215_MOESM3_ESM.zip › Figure 1 38/1L/Vinculin BAT thermoneutrality.png]

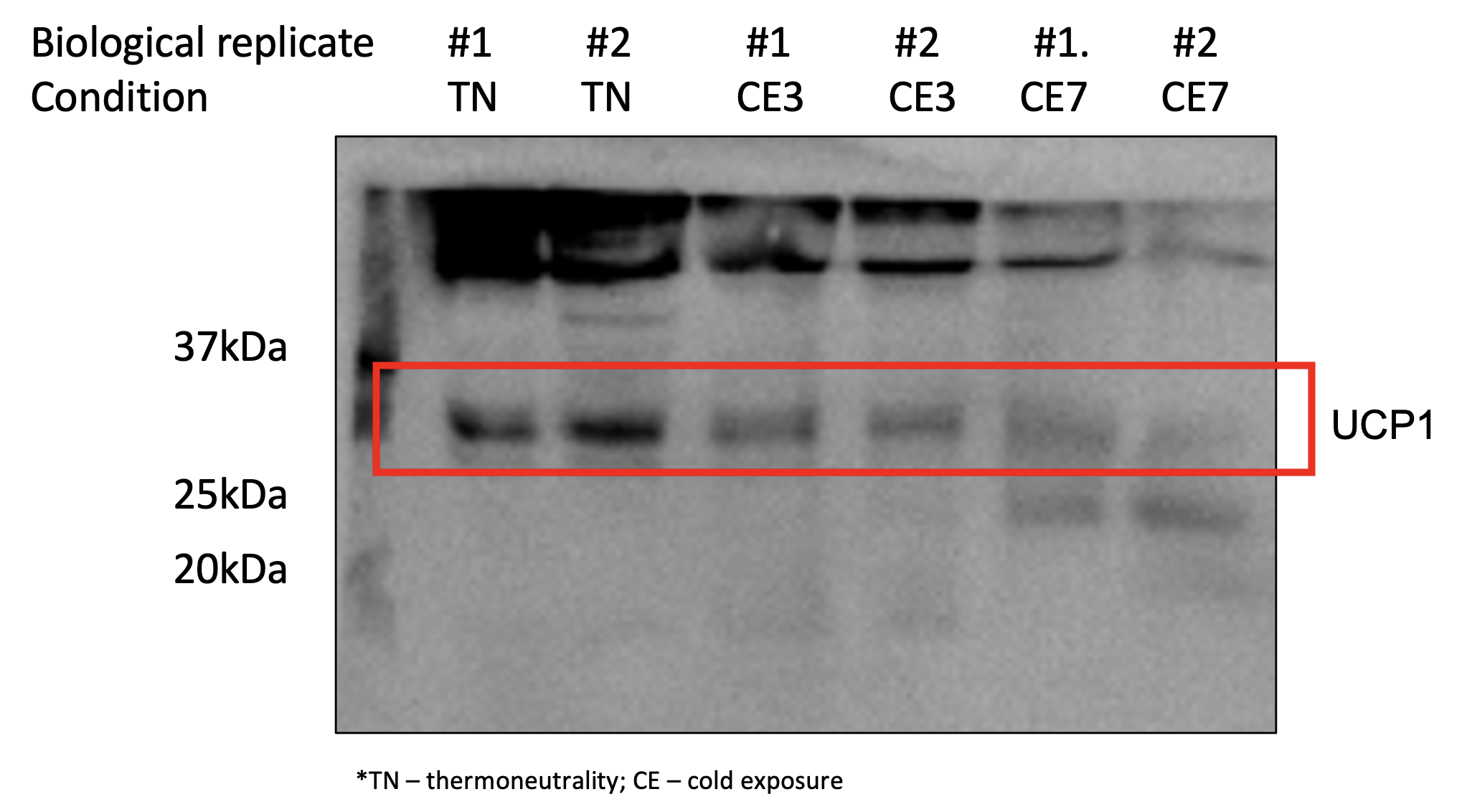

Supplement: Supplementary file 3 — Source data Fig. 1 [file 44318_2024_215_MOESM3_ESM.zip › Figure 1 38/1L/UCP1 BAT thermoneutrality.png]

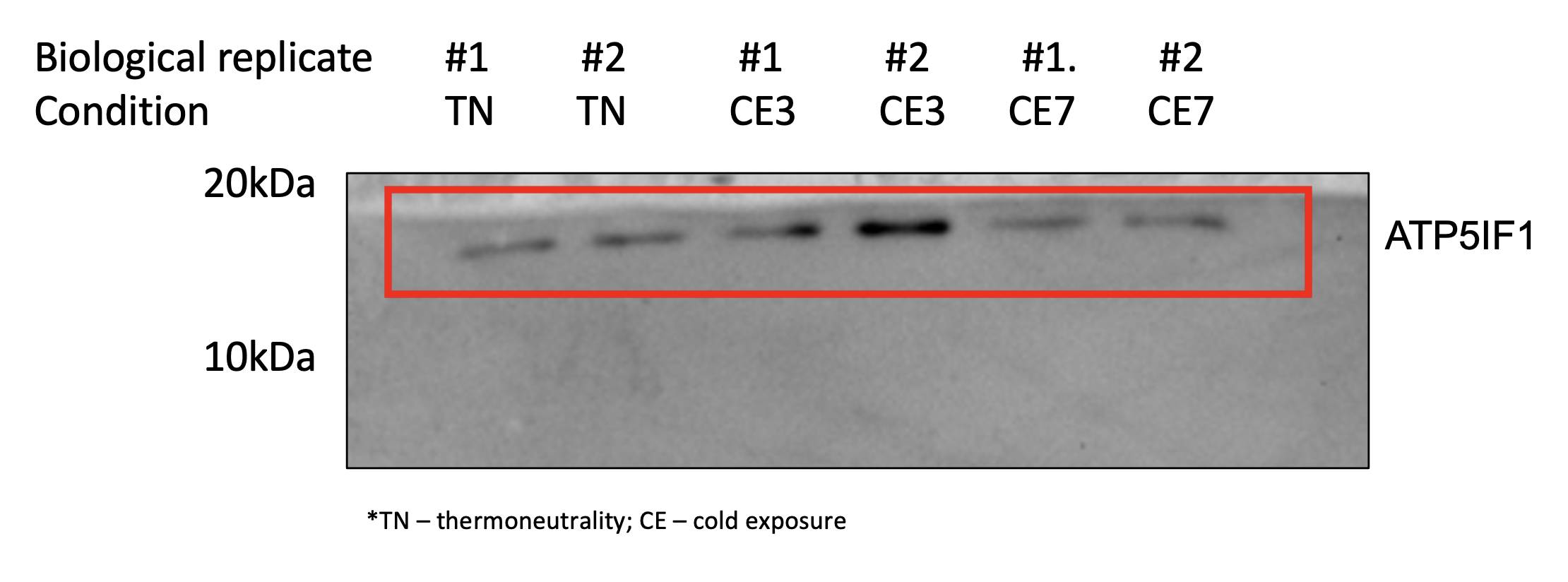

Supplement: Supplementary file 3 — Source data Fig. 1 [file 44318_2024_215_MOESM3_ESM.zip › Figure 1 38/1L/ATP5IF1 BAT thermoneutrality.png]

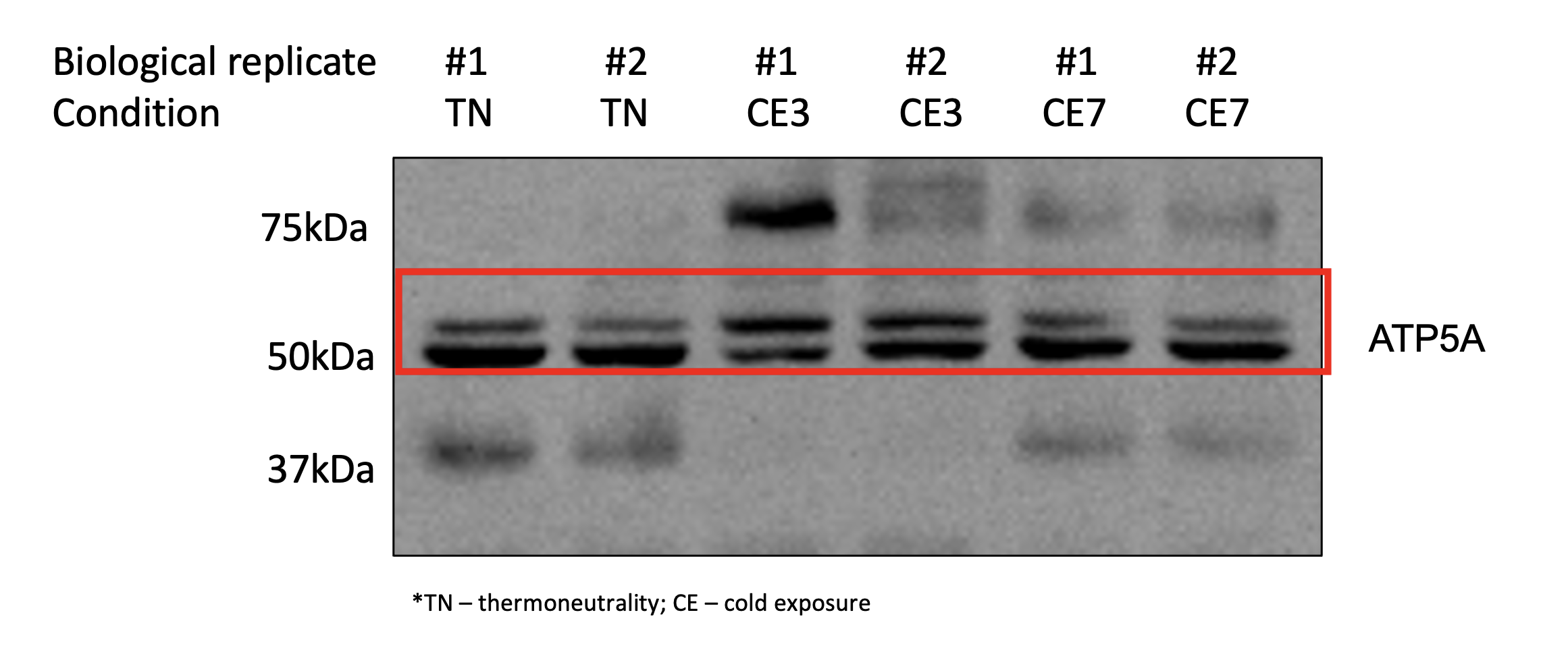

Supplement: Supplementary file 3 — Source data Fig. 1 [file 44318_2024_215_MOESM3_ESM.zip › Figure 1 38/1L/Complex V BAT thermoneutrality.png]

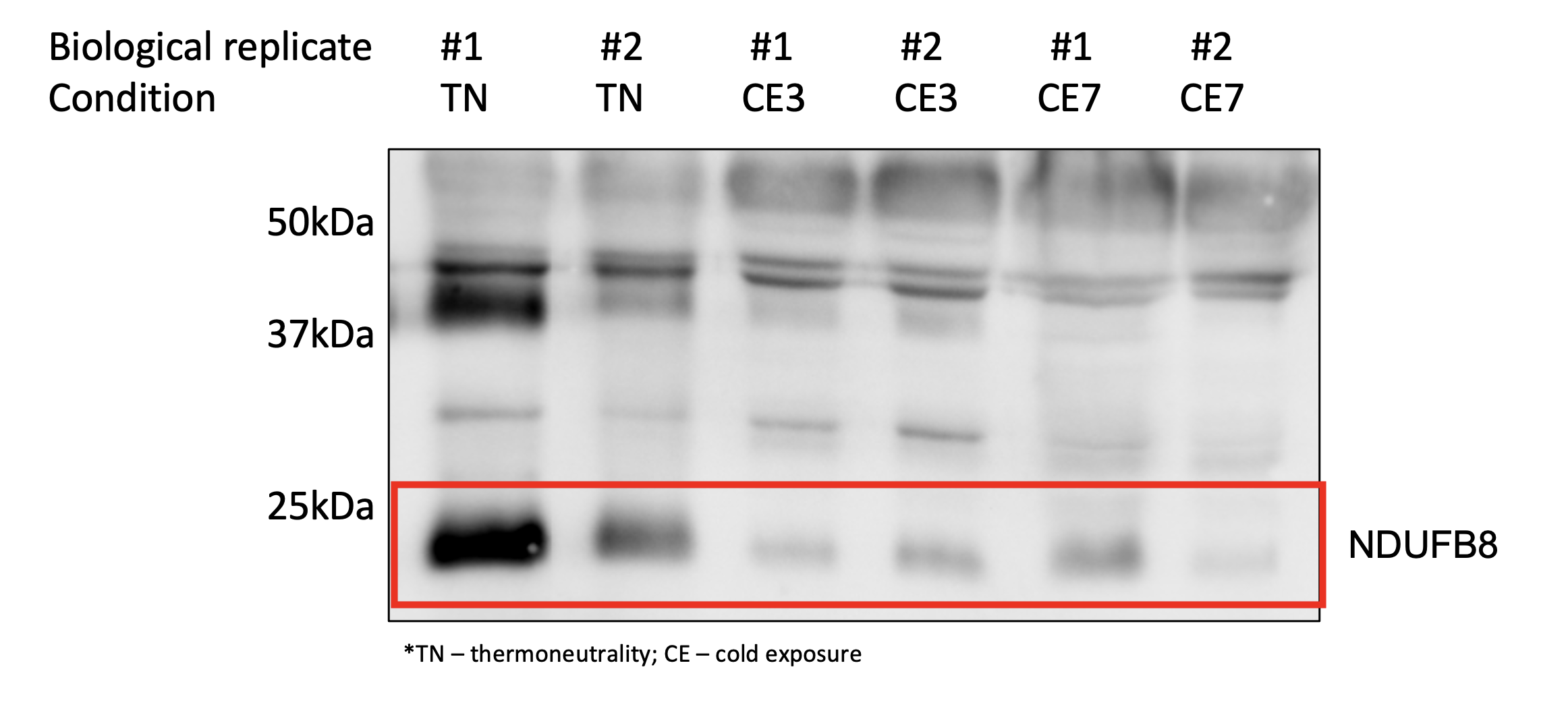

Supplement: Supplementary file 3 — Source data Fig. 1 [file 44318_2024_215_MOESM3_ESM.zip › Figure 1 38/1L/Complex I BAT thermoneutrality.png]

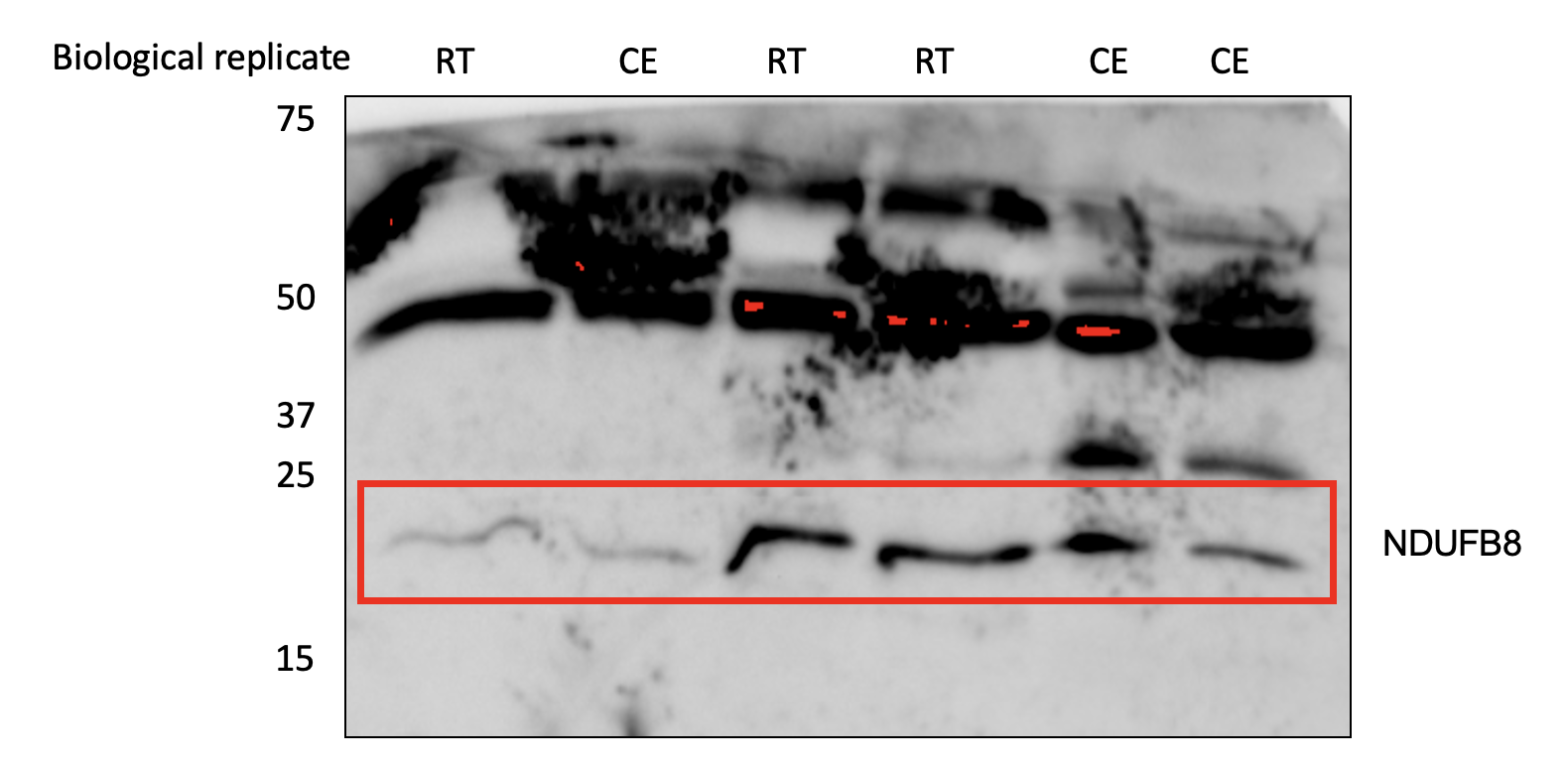

Supplement: Supplementary file 3 — Source data Fig. 1 [file 44318_2024_215_MOESM3_ESM.zip › Figure 1 38/1C/NDUFB8.png]

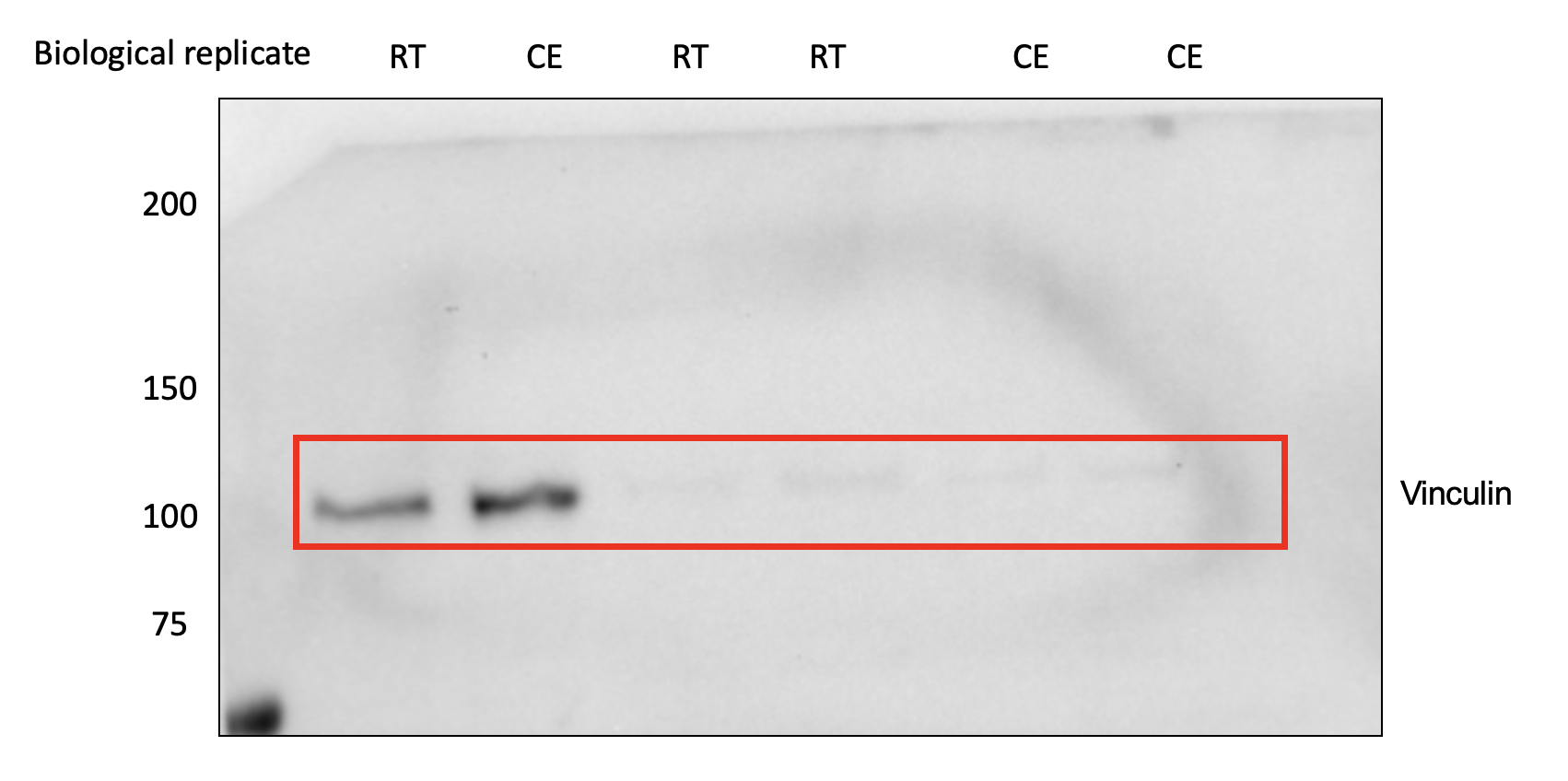

Supplement: Supplementary file 3 — Source data Fig. 1 [file 44318_2024_215_MOESM3_ESM.zip › Figure 1 38/1C/Vinculin.png]

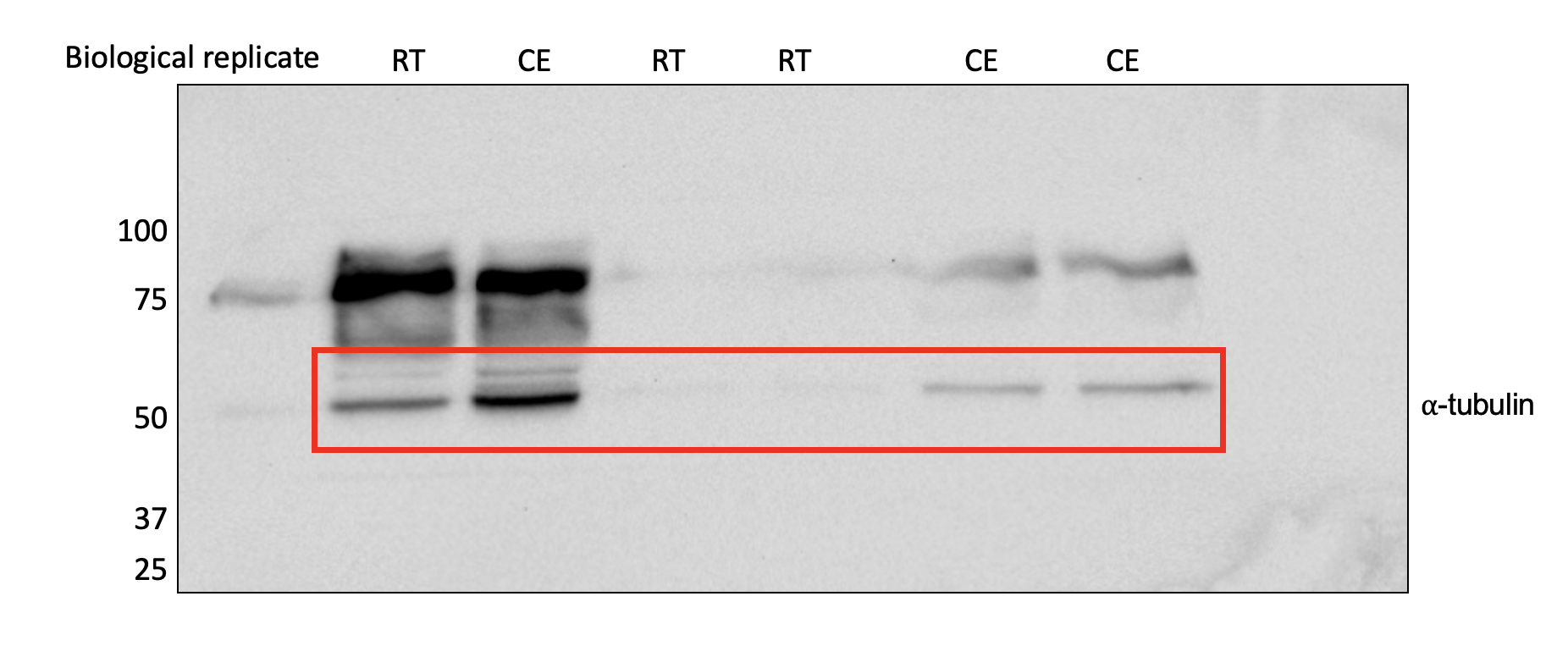

Supplement: Supplementary file 3 — Source data Fig. 1 [file 44318_2024_215_MOESM3_ESM.zip › Figure 1 38/1C/alpha-tubulin.png]

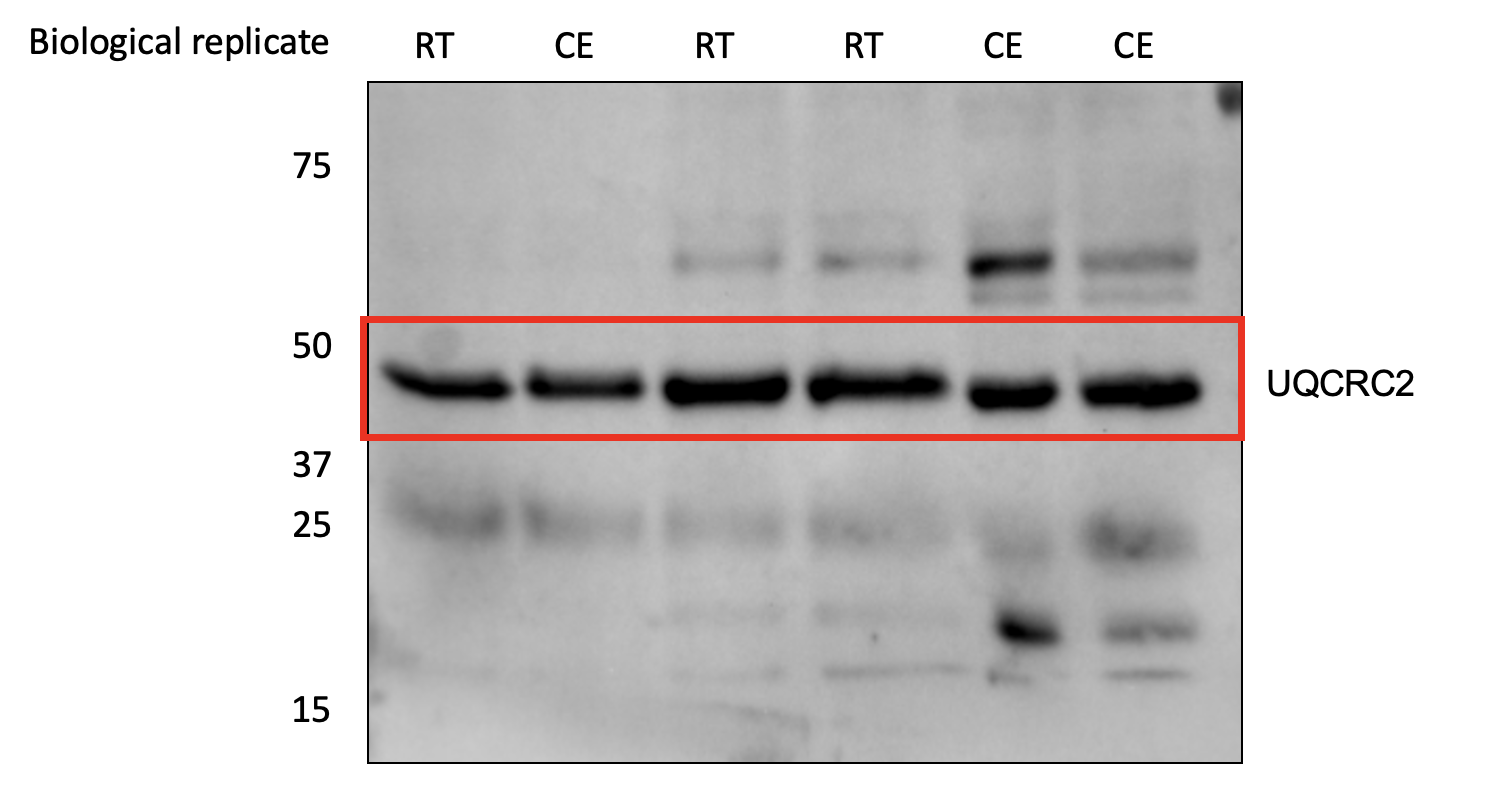

Supplement: Supplementary file 3 — Source data Fig. 1 [file 44318_2024_215_MOESM3_ESM.zip › Figure 1 38/1C/UQCRC2.png]

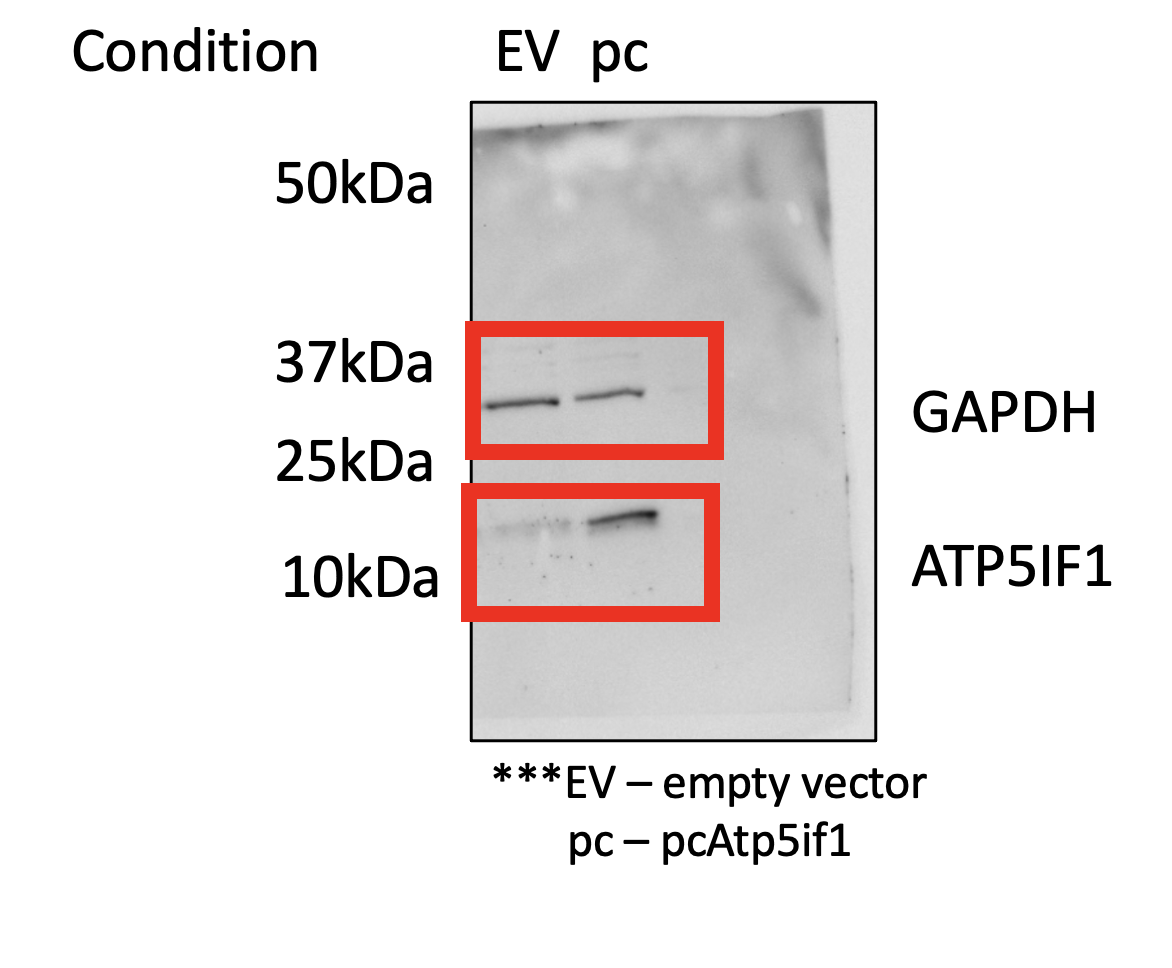

Supplement: Supplementary file 4 — Source data Fig. 2 [file 44318_2024_215_MOESM4_ESM.zip › Figure 2/2G/IF1 overexpression in brown adipocytes.png]

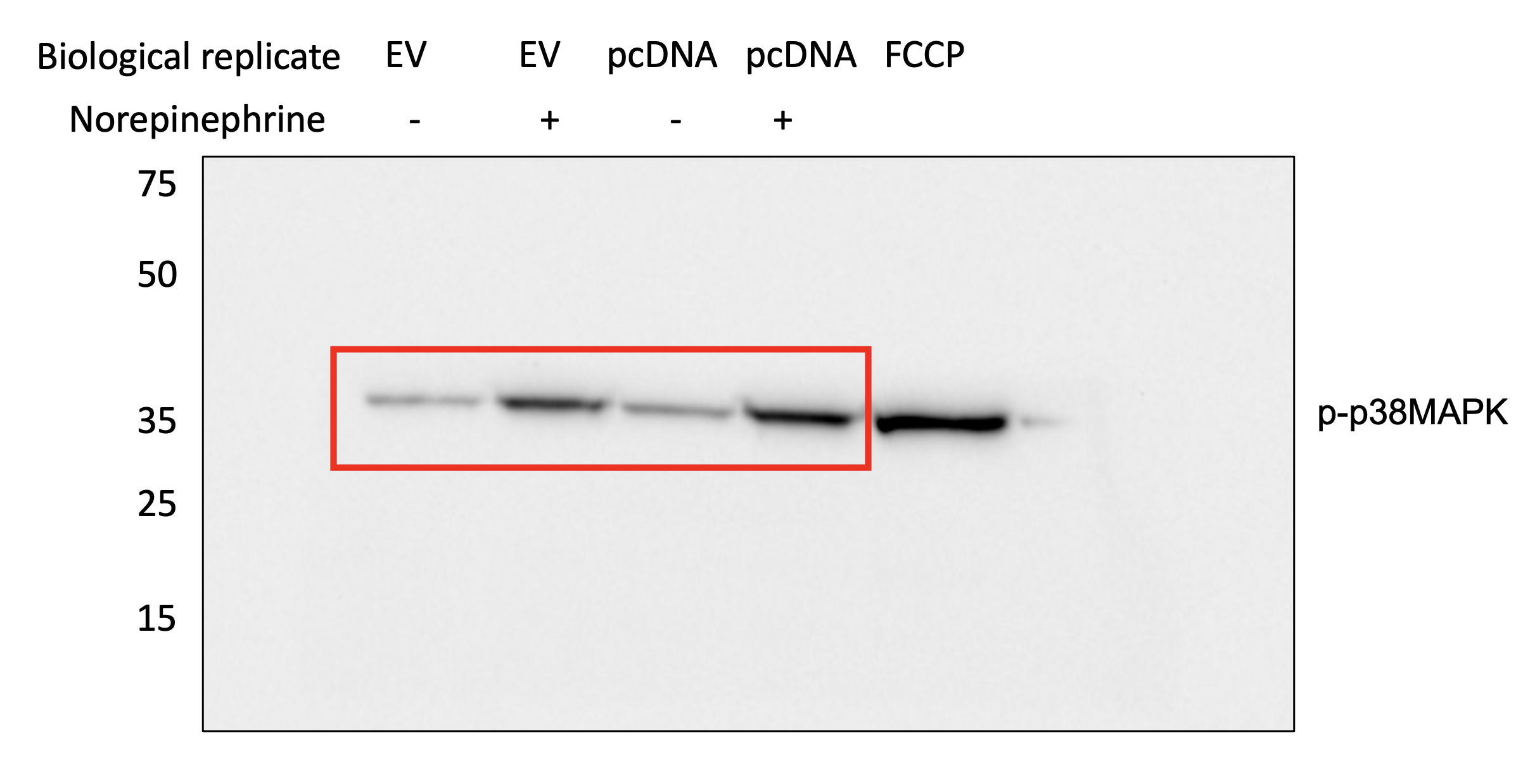

Supplement: Supplementary file 4 — Source data Fig. 2 [file 44318_2024_215_MOESM4_ESM.zip › Figure 2/2H/p-p38MAPK.png]

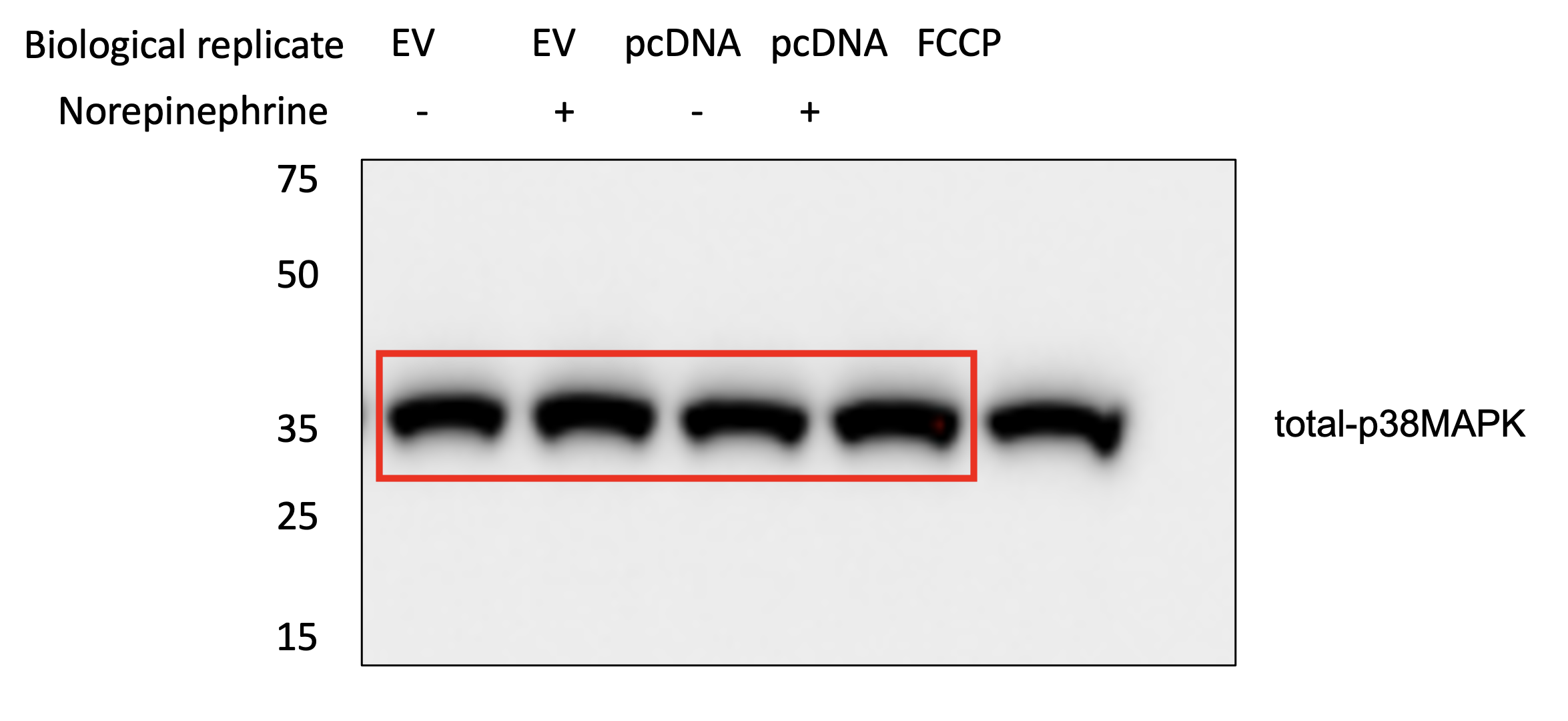

Supplement: Supplementary file 4 — Source data Fig. 2 [file 44318_2024_215_MOESM4_ESM.zip › Figure 2/2H/total-p38MAPK.png]

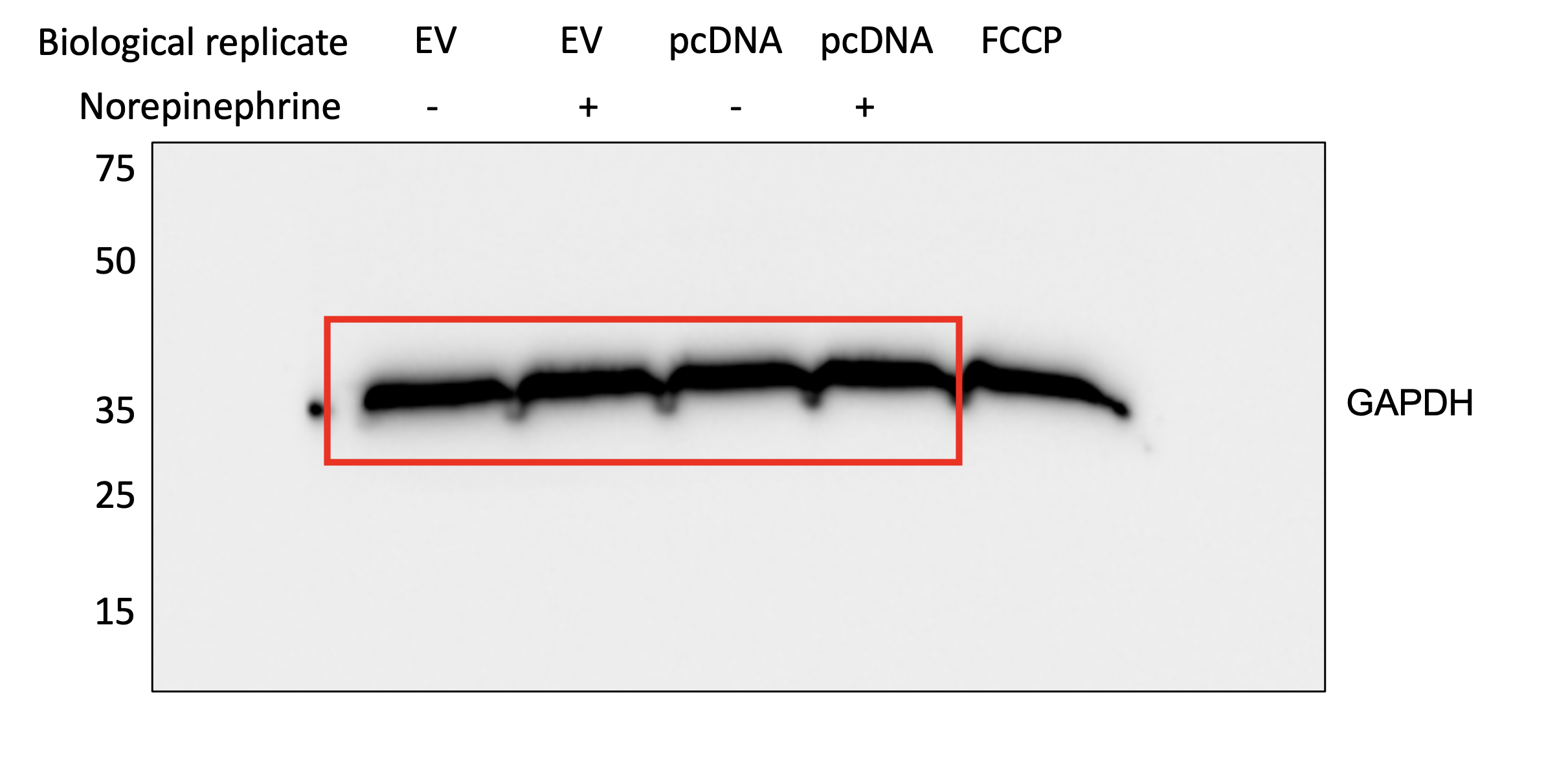

Supplement: Supplementary file 4 — Source data Fig. 2 [file 44318_2024_215_MOESM4_ESM.zip › Figure 2/2H/GAPDH.png]

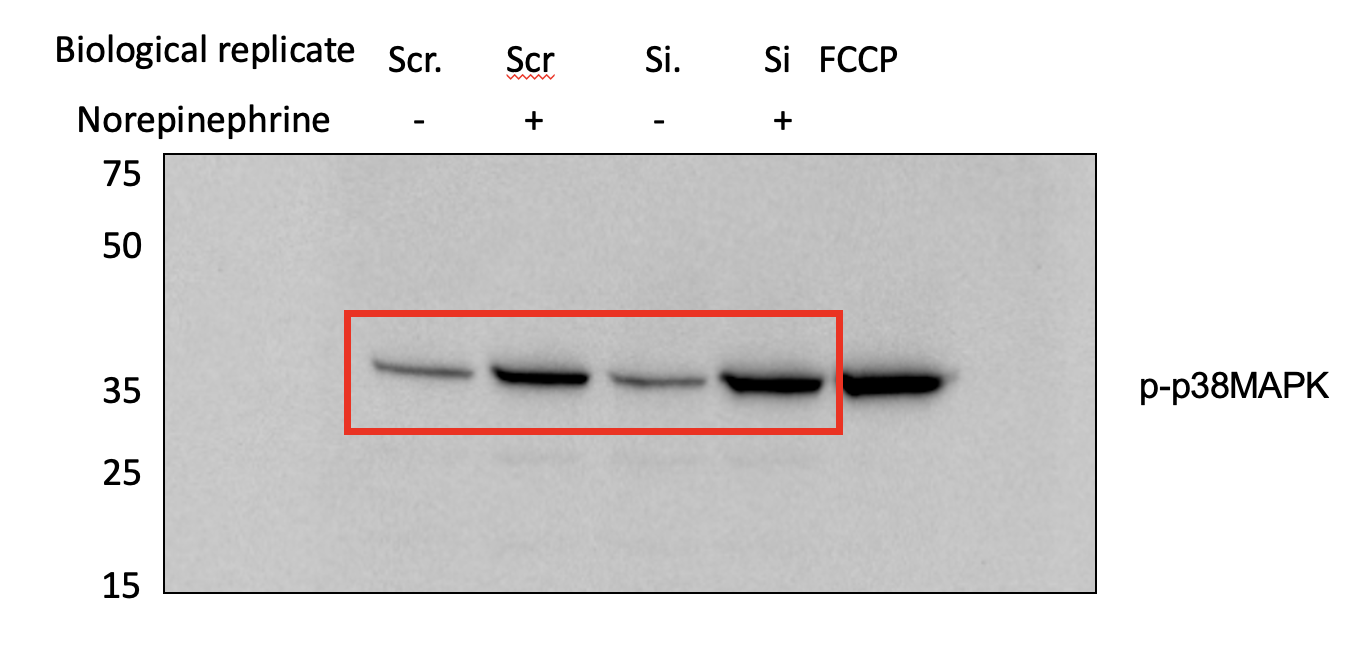

Supplement: Supplementary file 4 — Source data Fig. 2 [file 44318_2024_215_MOESM4_ESM.zip › Figure 2/2C/p-p38MAPK.png]

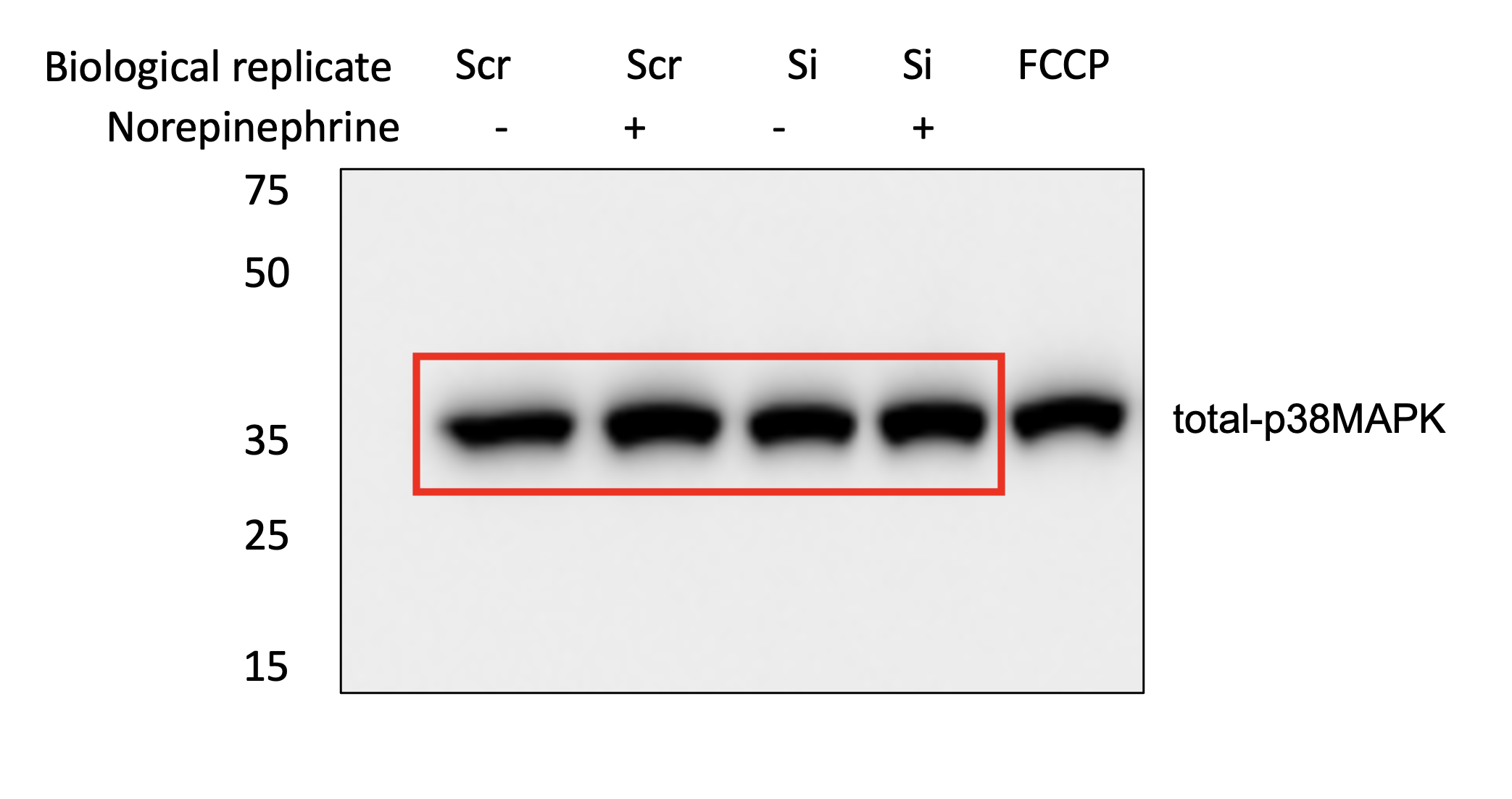

Supplement: Supplementary file 4 — Source data Fig. 2 [file 44318_2024_215_MOESM4_ESM.zip › Figure 2/2C/total-p38MAPK.png]

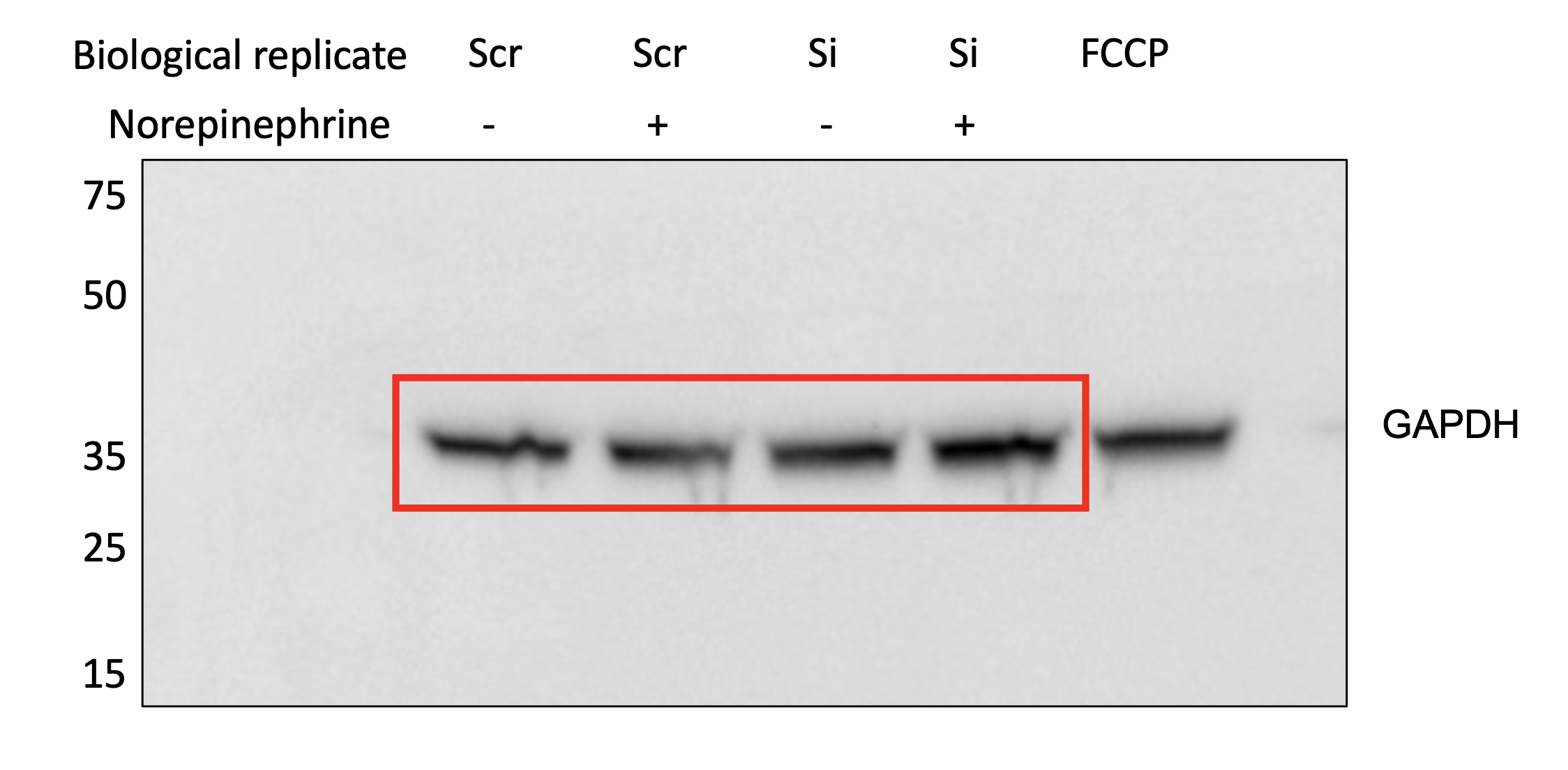

Supplement: Supplementary file 4 — Source data Fig. 2 [file 44318_2024_215_MOESM4_ESM.zip › Figure 2/2C/GAPDH.png]

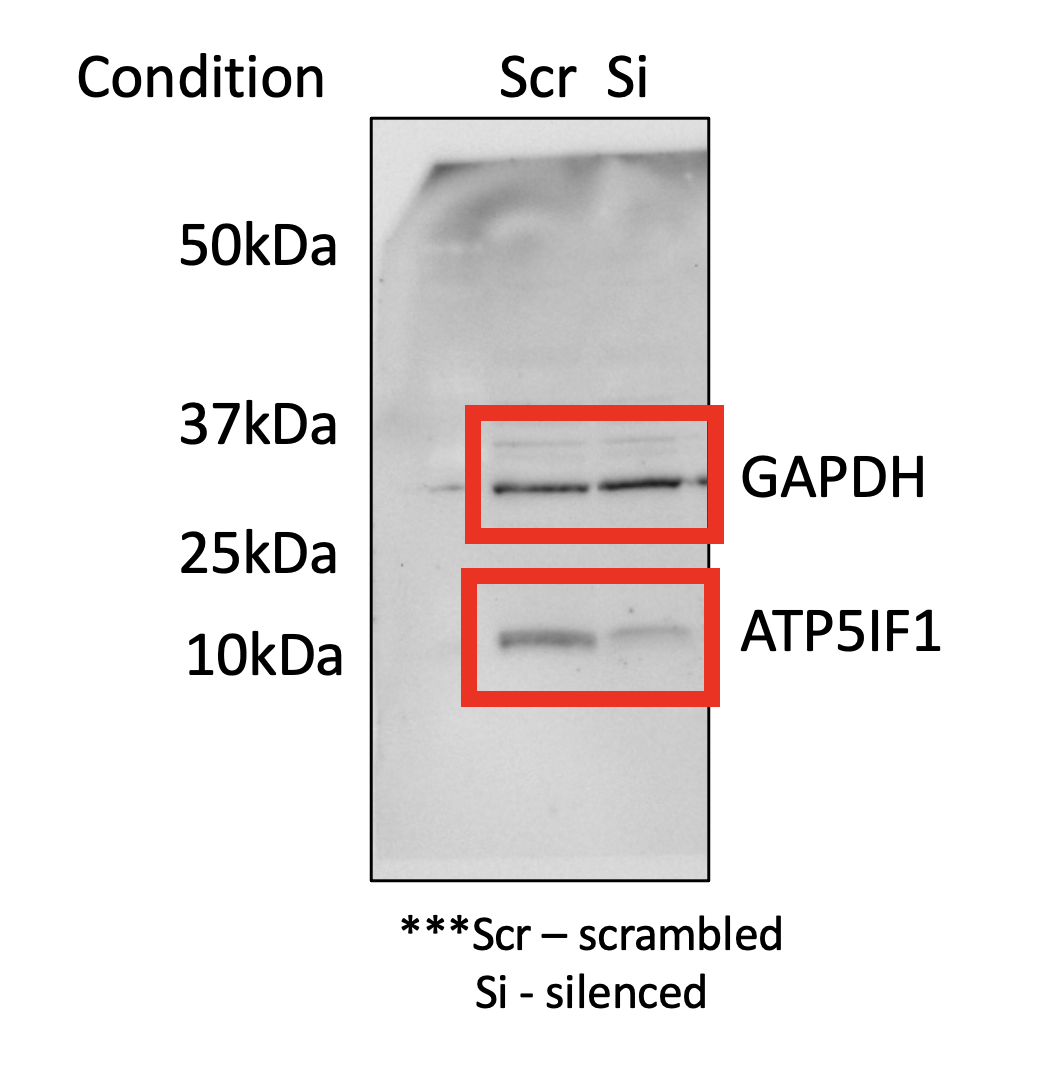

Supplement: Supplementary file 4 — Source data Fig. 2 [file 44318_2024_215_MOESM4_ESM.zip › Figure 2/2B/IF1 silencing brown adipocytes.png]

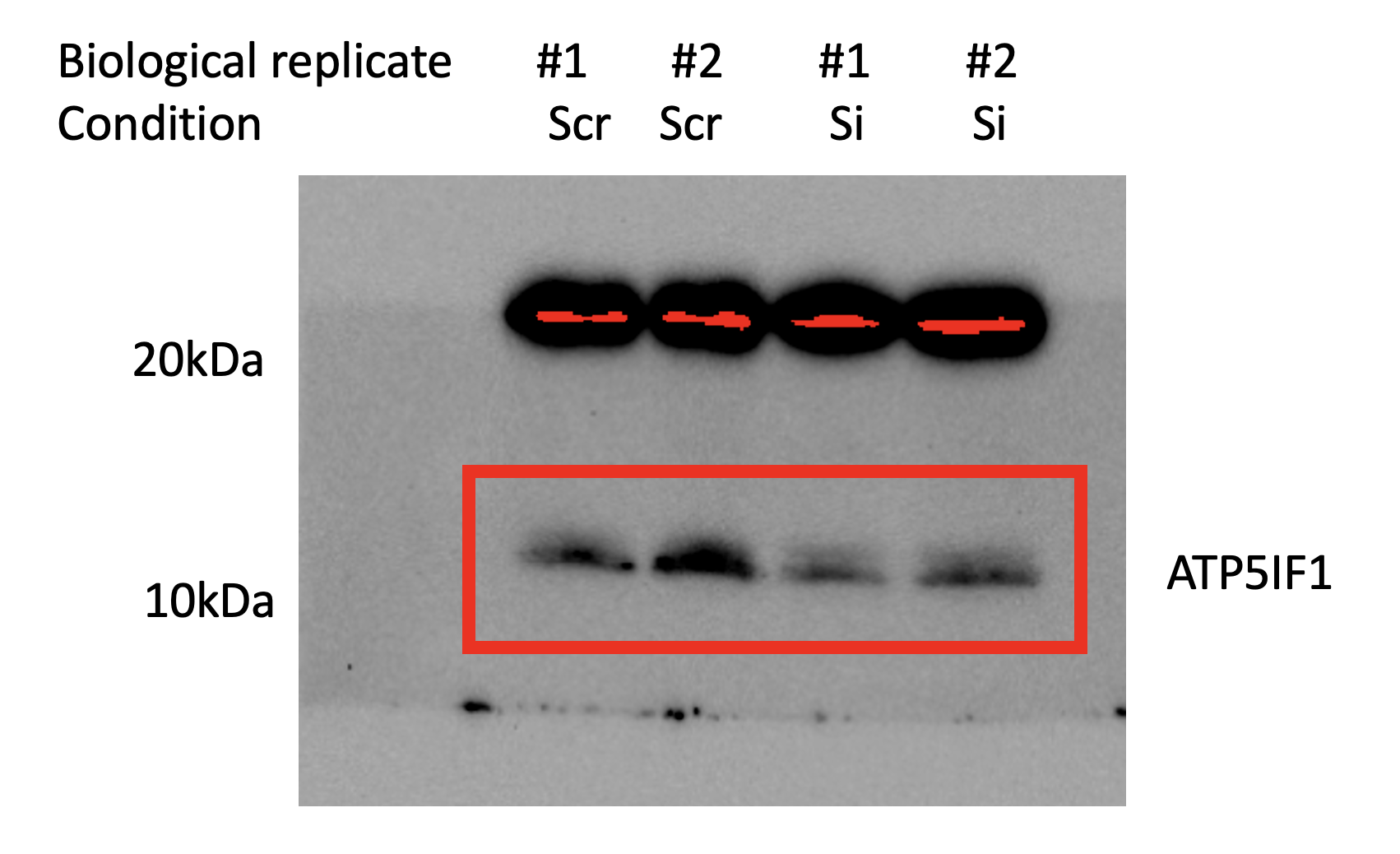

Supplement: Supplementary file 5 — Source data Fig. 3 [file 44318_2024_215_MOESM5_ESM.zip › Figure 3/3B/ATP5IF1 knockdown.png]

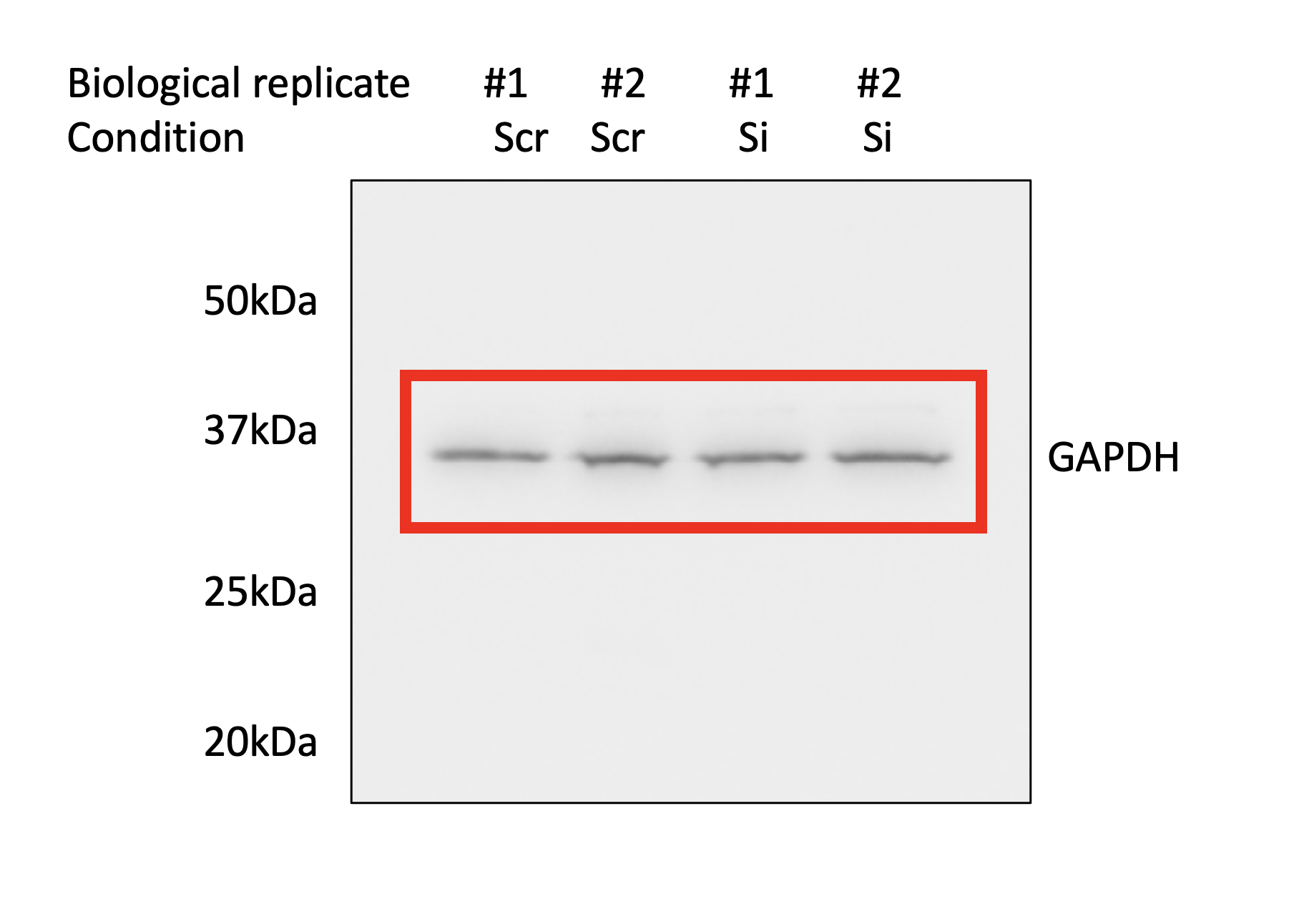

Supplement: Supplementary file 5 — Source data Fig. 3 [file 44318_2024_215_MOESM5_ESM.zip › Figure 3/3B/GAPDH IF1 knockdown.png]

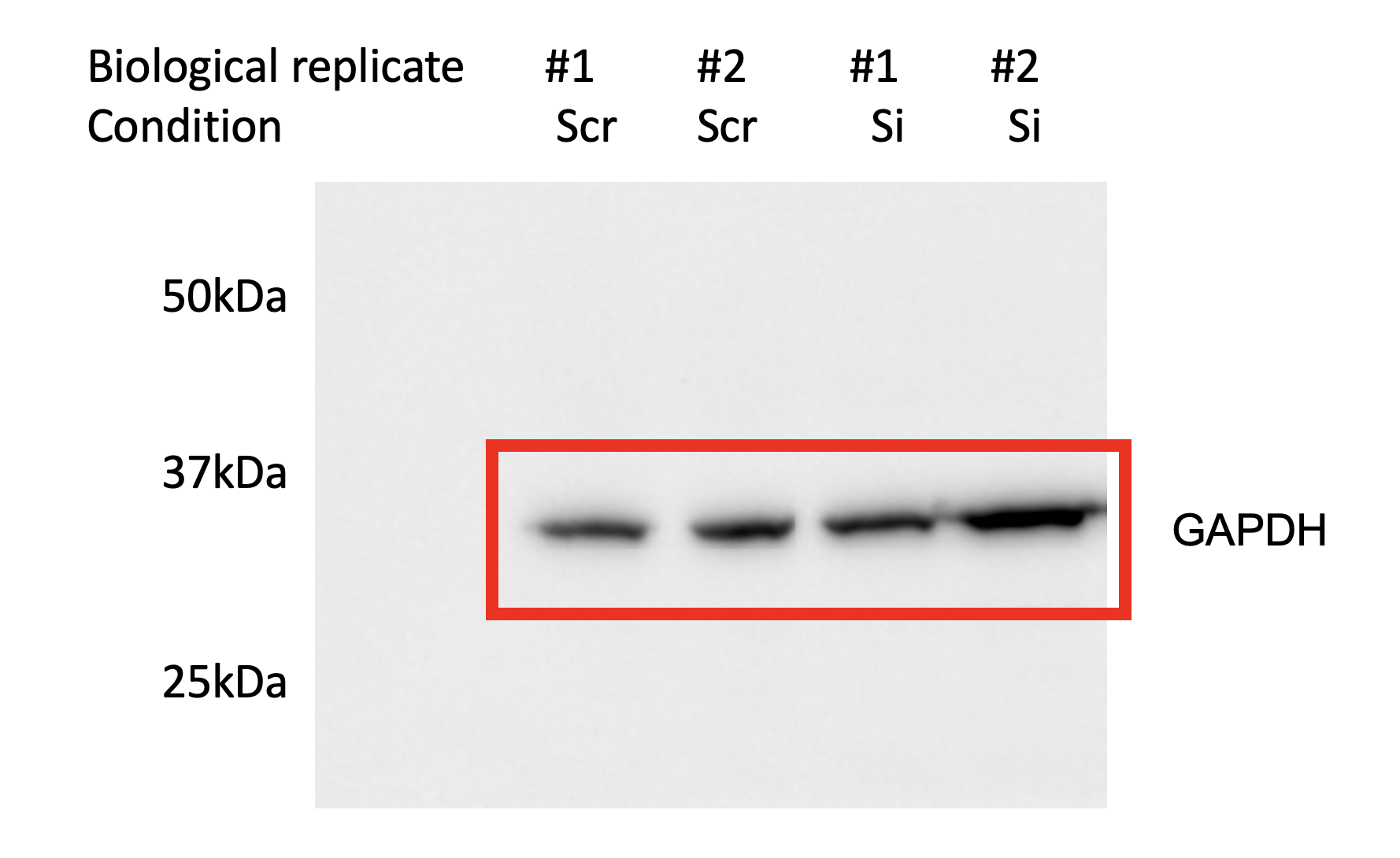

Supplement: Supplementary file 5 — Source data Fig. 3 [file 44318_2024_215_MOESM5_ESM.zip › Figure 3/3G/GAPDH if1 knockdown.png]

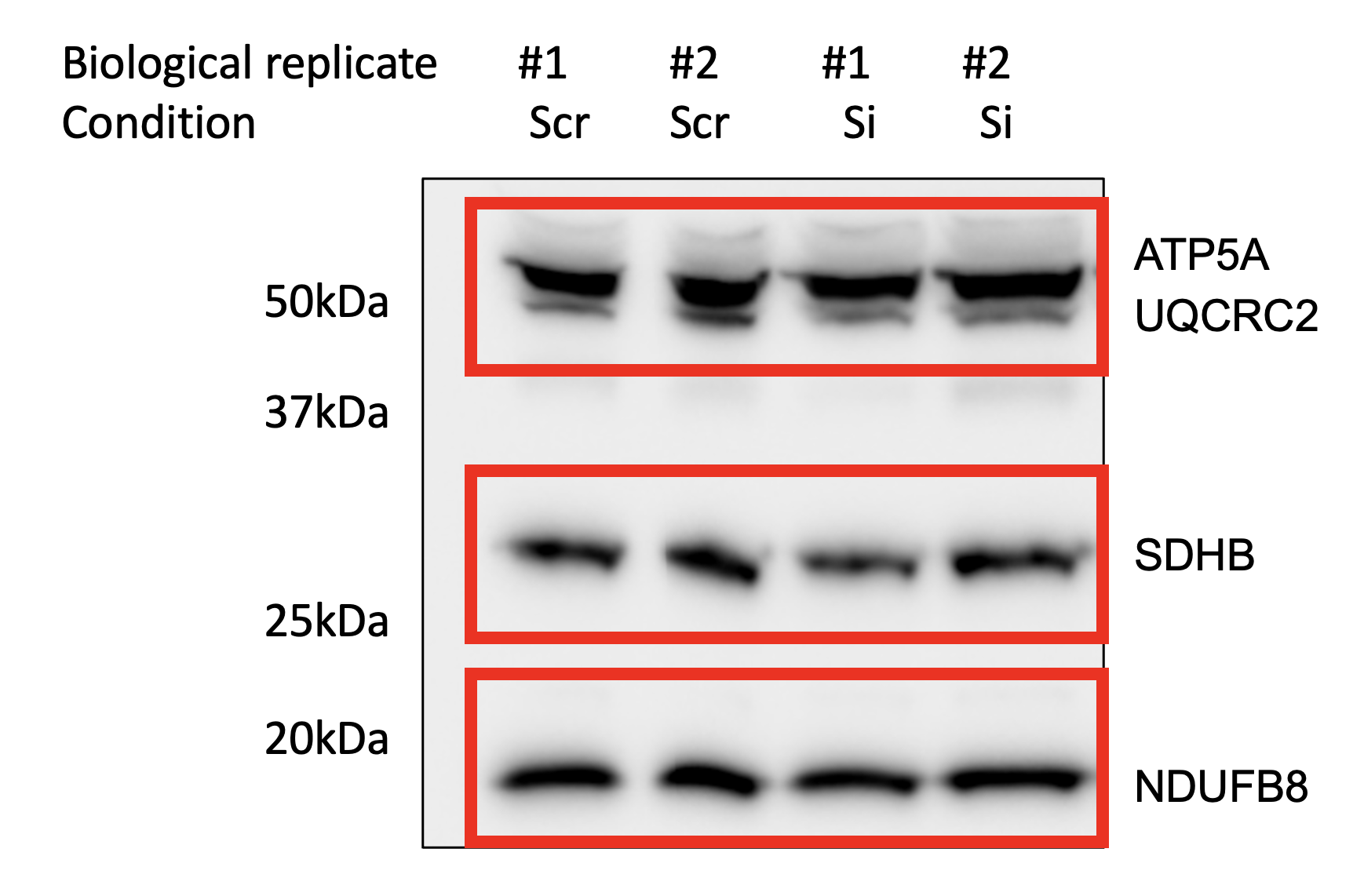

Supplement: Supplementary file 5 — Source data Fig. 3 [file 44318_2024_215_MOESM5_ESM.zip › Figure 3/3G/OXPHOS if1 knockdown.png]

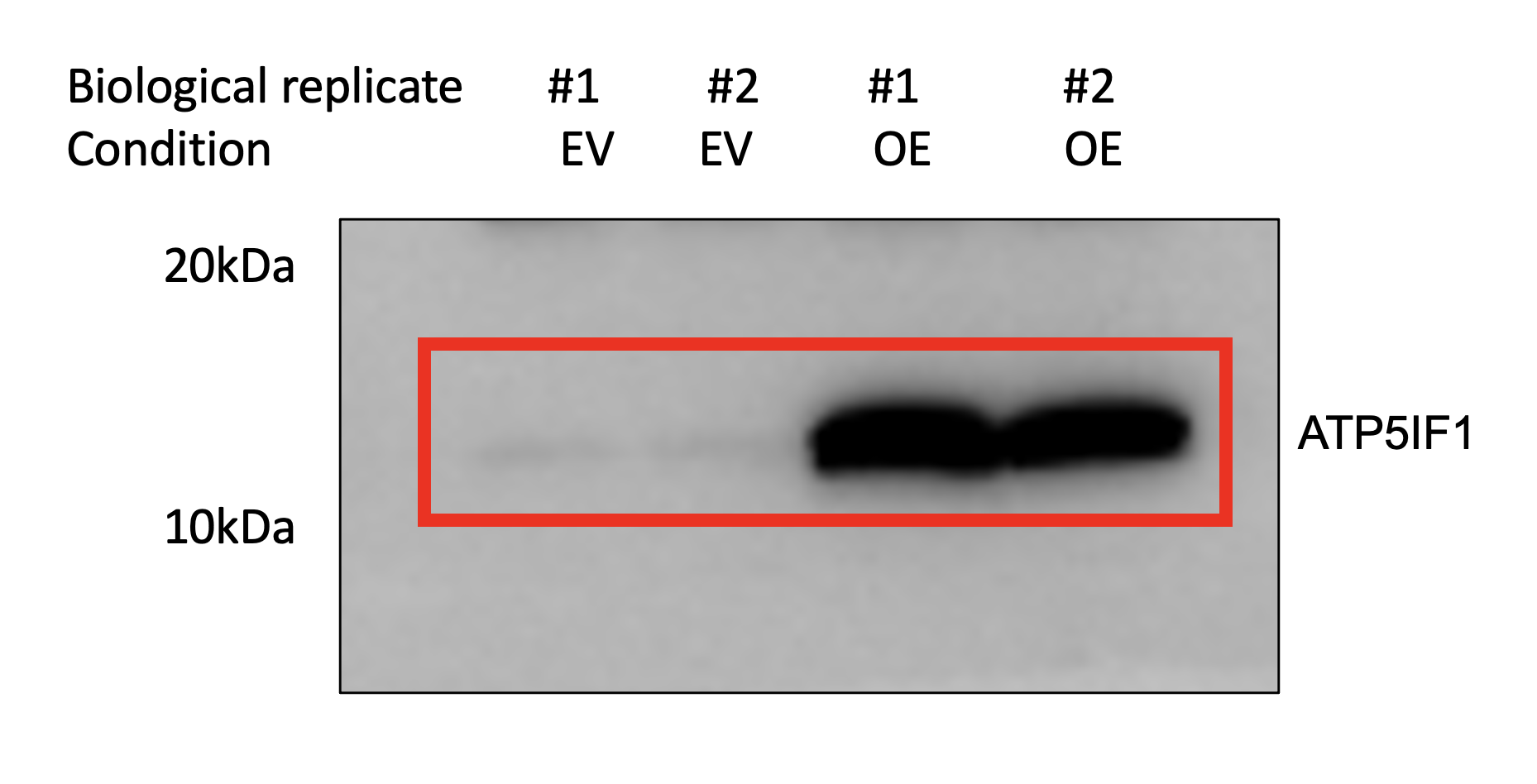

Supplement: Supplementary file 6 — Source data Fig. 4 [file 44318_2024_215_MOESM6_ESM.zip › Figure 4/4B/ATP5IF1 overexpression.png]

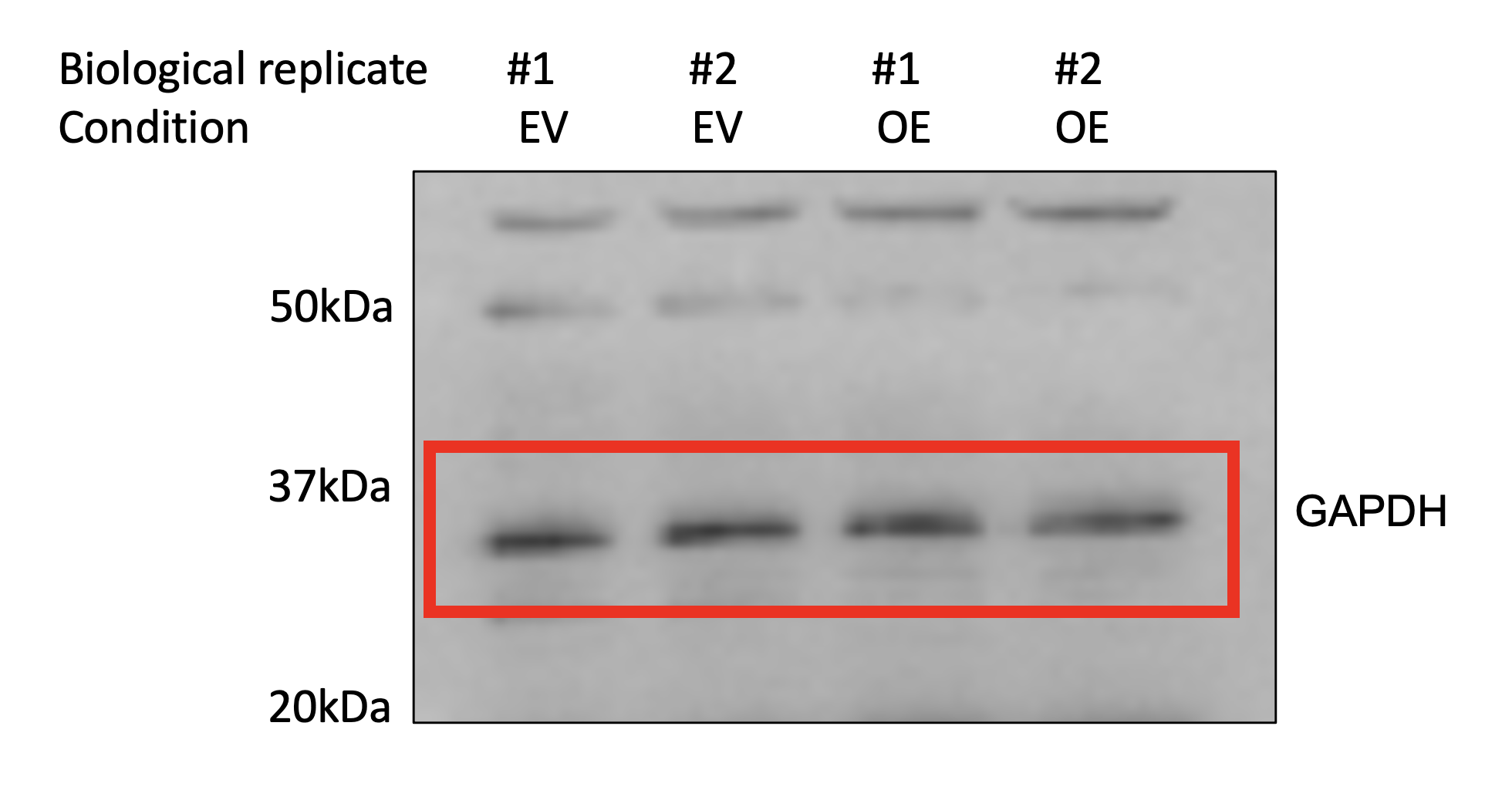

Supplement: Supplementary file 6 — Source data Fig. 4 [file 44318_2024_215_MOESM6_ESM.zip › Figure 4/4B/GAPDH atp5if1 overexpression.png]

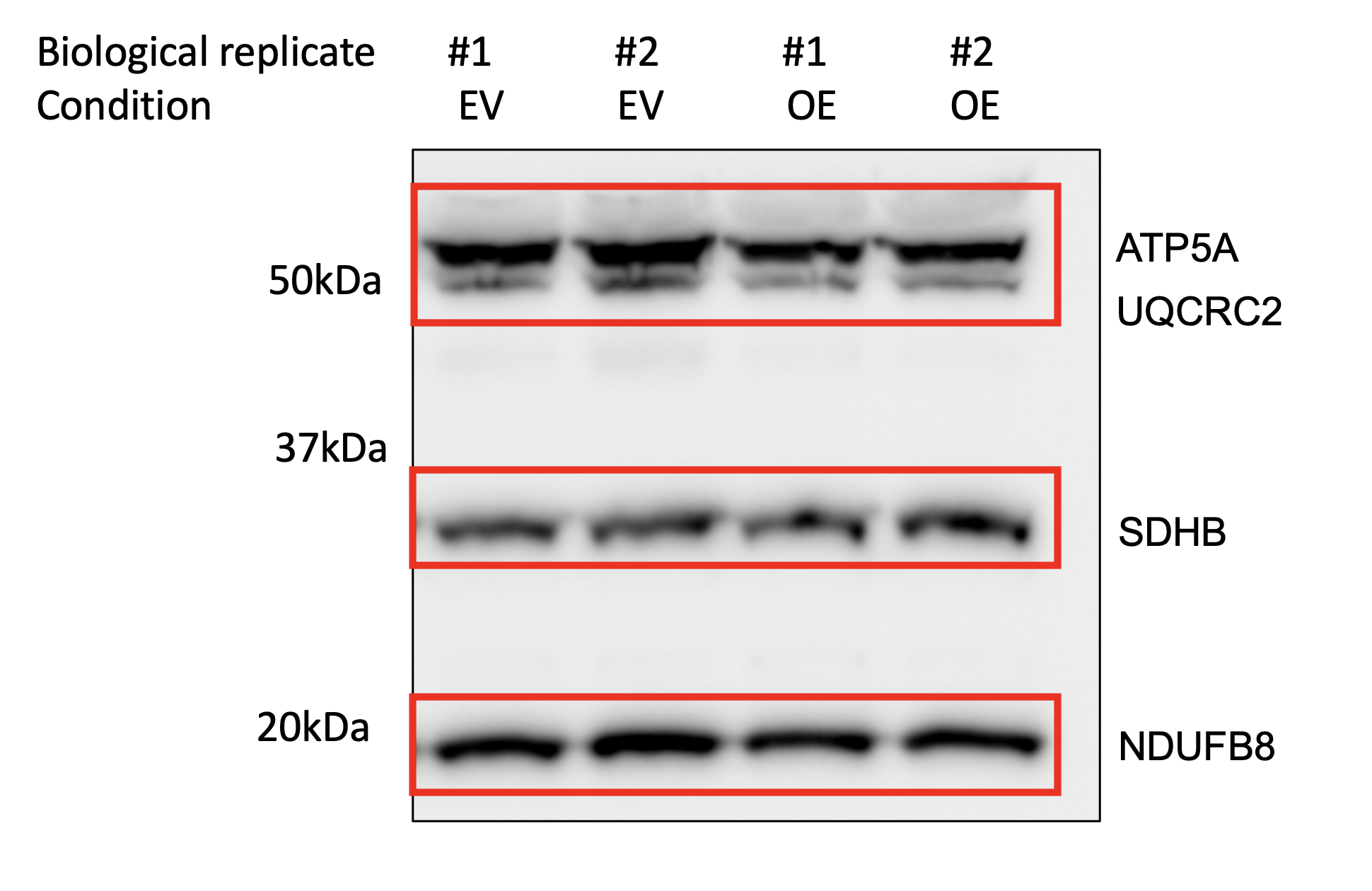

Supplement: Supplementary file 6 — Source data Fig. 4 [file 44318_2024_215_MOESM6_ESM.zip › Figure 4/4I/OXPHOS if1 overexpression.png]

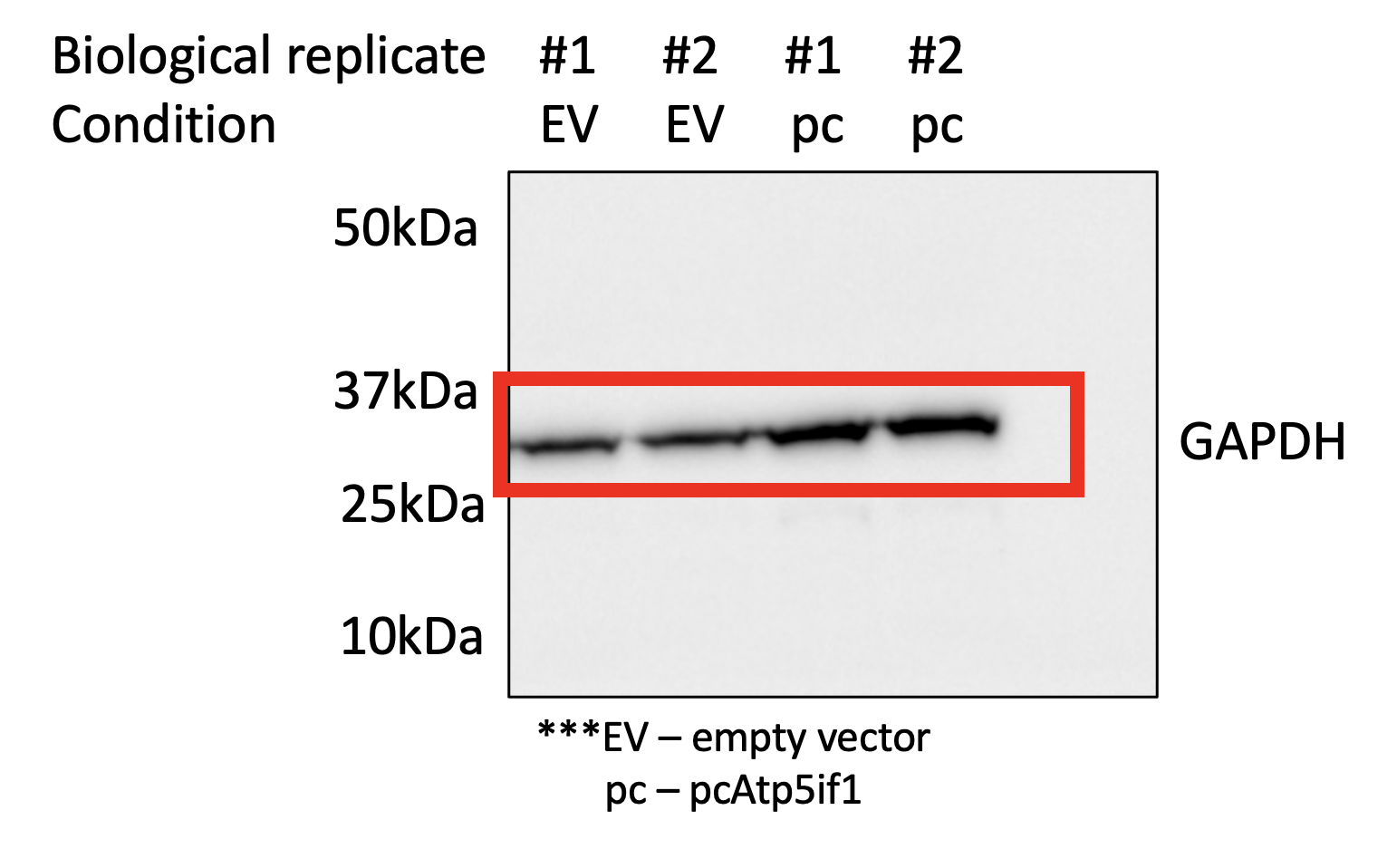

Supplement: Supplementary file 6 — Source data Fig. 4 [file 44318_2024_215_MOESM6_ESM.zip › Figure 4/4I/IF1 overexpression GAPDH.png]

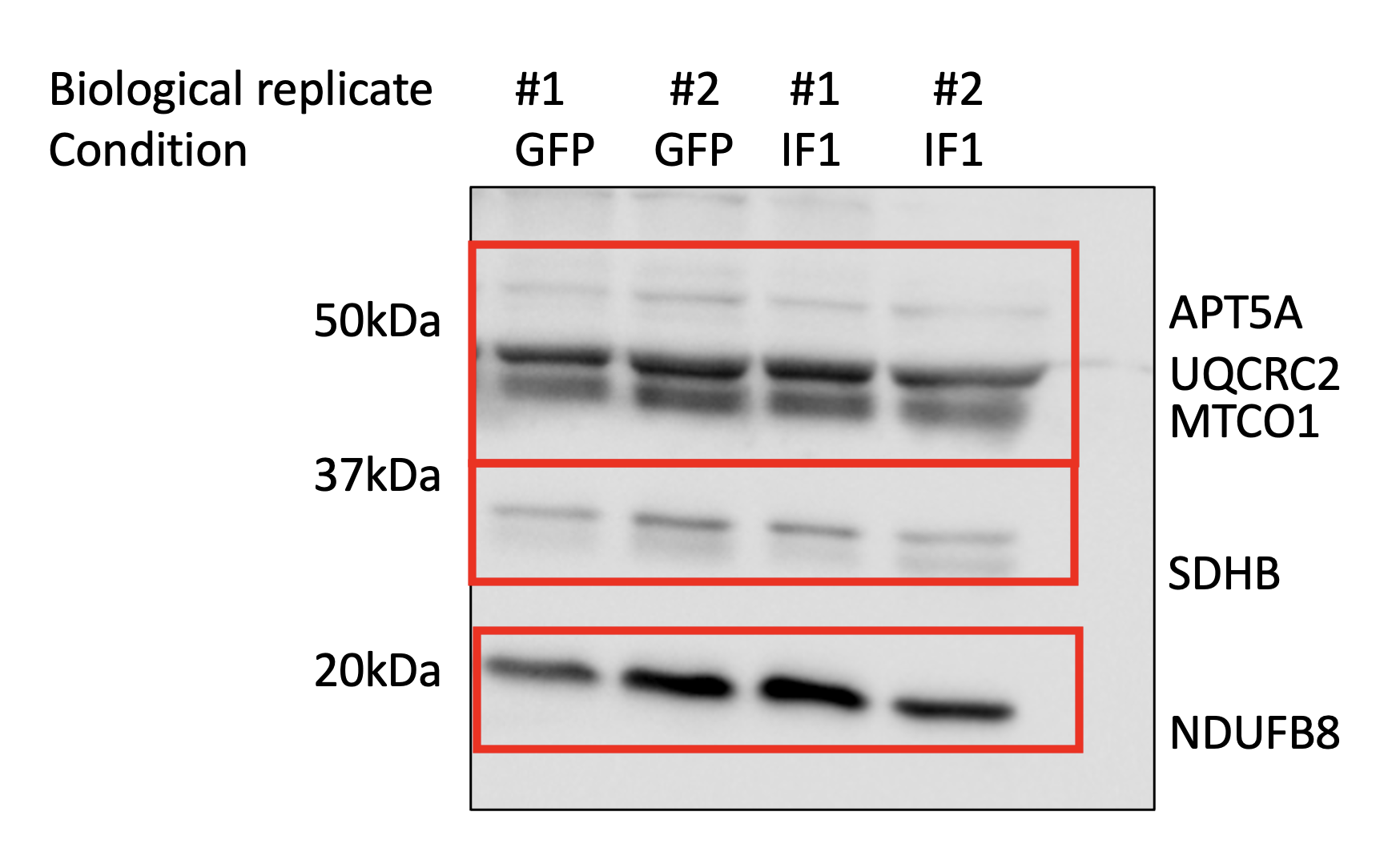

Supplement: Supplementary file 7 — Source data Fig. 5 [file 44318_2024_215_MOESM7_ESM.zip › Figure 5/5J/OXPHOS AAV-IF1 BAT.png]

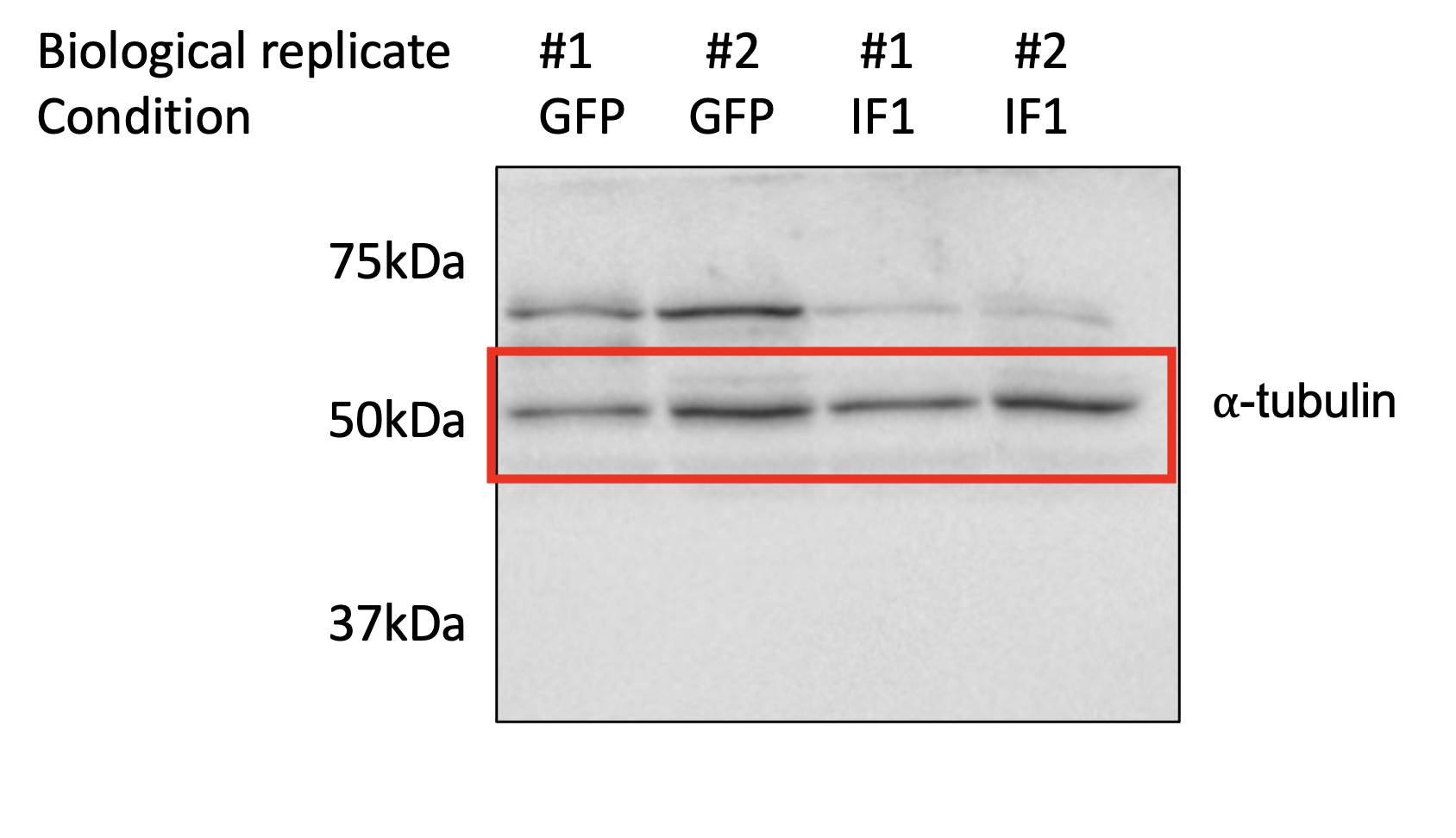

Supplement: Supplementary file 7 — Source data Fig. 5 [file 44318_2024_215_MOESM7_ESM.zip › Figure 5/5J/alpha-tubulin AAV-IF1 BAT.png]

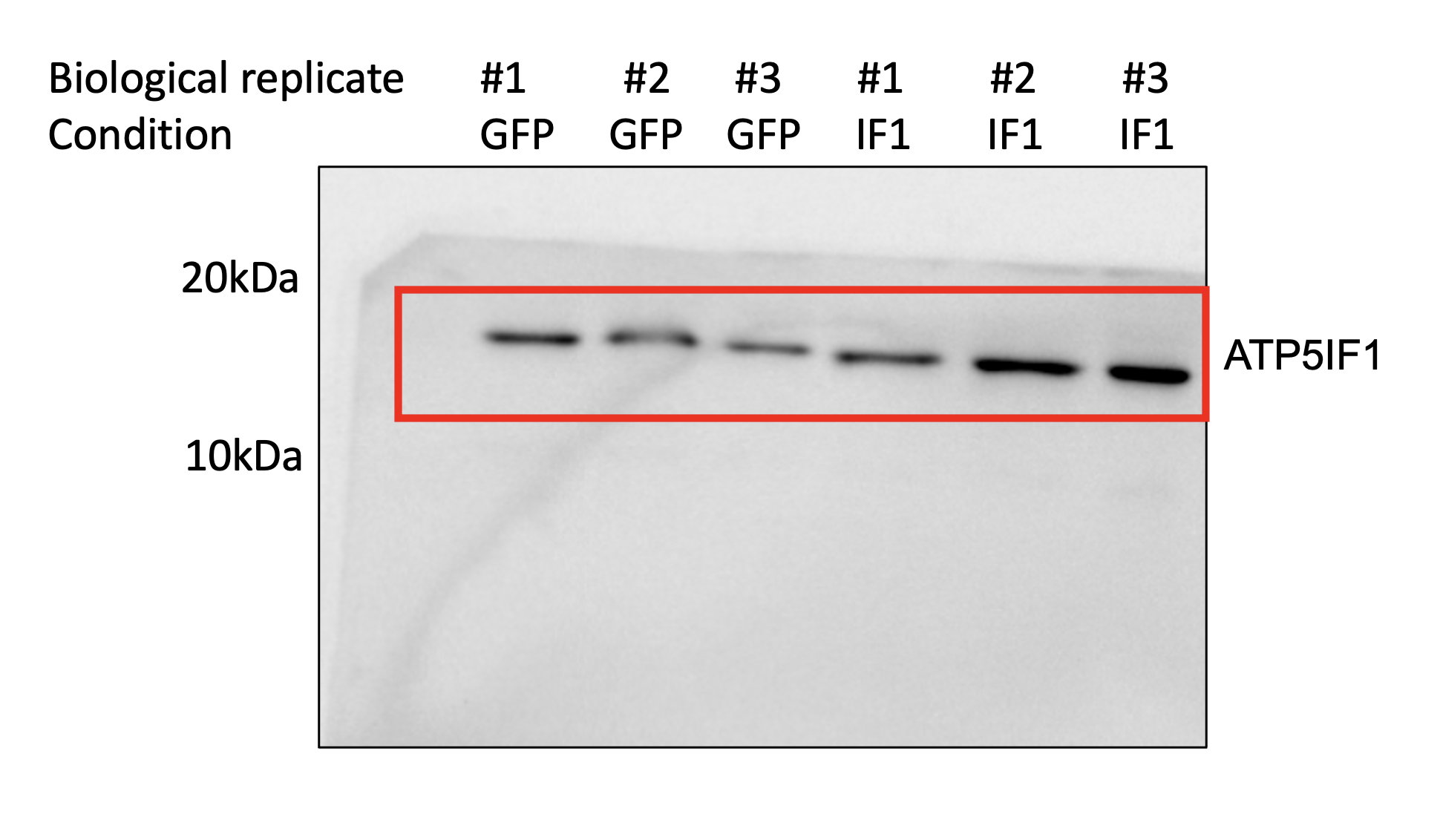

Supplement: Supplementary file 7 — Source data Fig. 5 [file 44318_2024_215_MOESM7_ESM.zip › Figure 5/5C/ATPIF1 AAV-IF1 BAT.png]

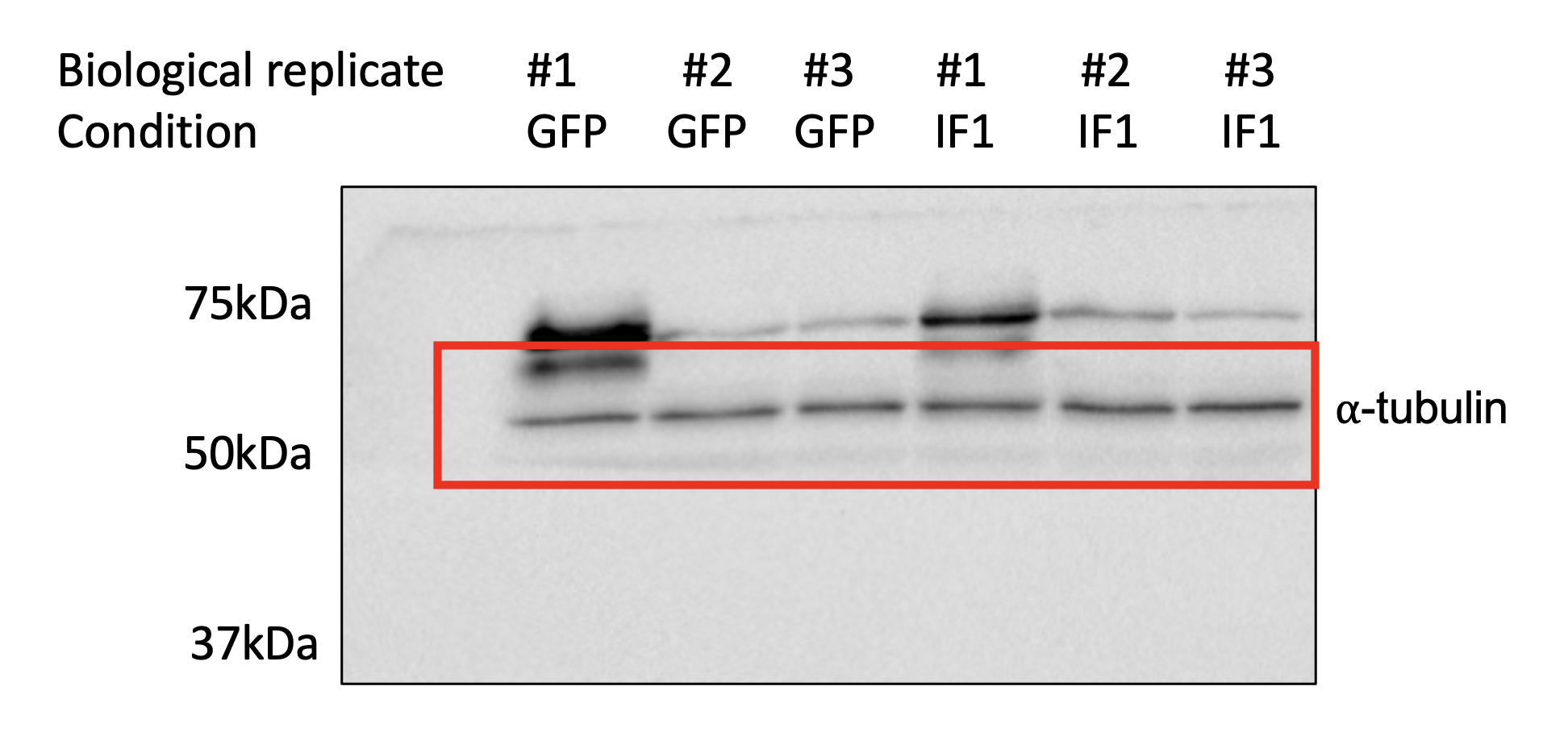

Supplement: Supplementary file 7 — Source data Fig. 5 [file 44318_2024_215_MOESM7_ESM.zip › Figure 5/5C/alpha-tubulin AAV-IF1 BAT.png]
