# Supplementary material for: IF1 is a cold-regulated switch of ATP synthase hydrolytic activity to support thermogenesis in brown fat
Source: EMBO J. 2024 Sep 16;43(21):6. doi: 10.1038/s44318-024-00215-0 (PMC11535227; doi:10.1038/s44318-024-00215-0)
Supplement: Supplementary file 8 — Expanded View Figures [file 44318_2024_215_MOESM8_ESM.pdf]

Expanded View Figures

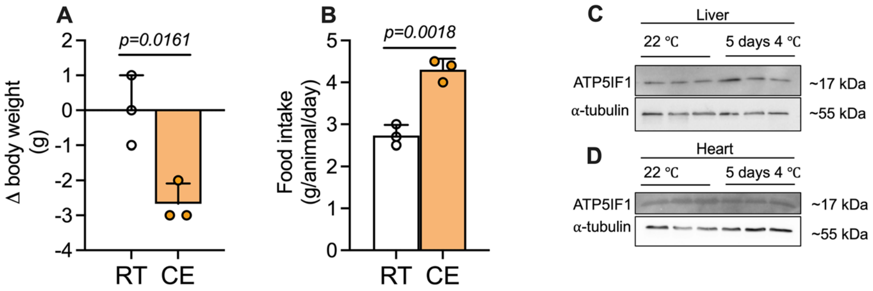

Figure EV1. Cold exposure induces body weight loss and does not alter IF1 levels in liver and heart.

(**A**) Change of body weight and (**B**) food intake during 5 days of cold exposure. IF1 protein levels in (**C**) liver and (**D**) heart after 5 days of cold exposure. IF1 - ATP synthase inhibitory factor subunit 1; Two-tailed Student's t-test (**A**, **B**). Data are expressed with individual values and mean ± SD superimposed. The exact p-value is displayed when $p < 0.05$.

© The Author(s)

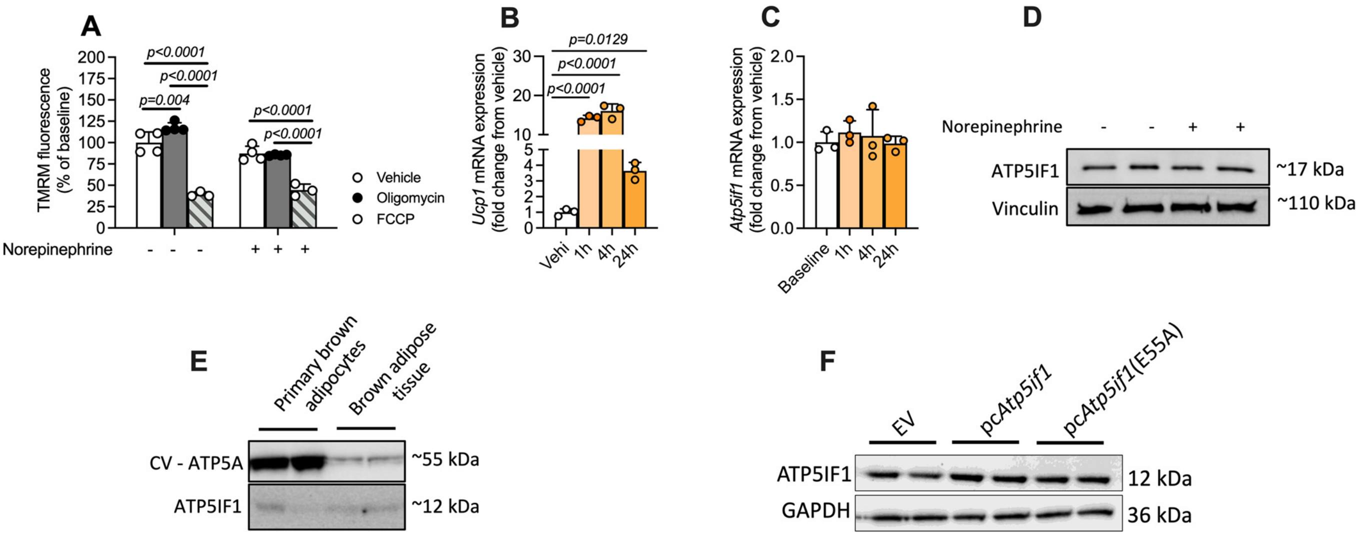

Figure EV2. Effects of oligomycin and FCCP on mitochondrial membrane potential.

(**A**) Brown adipocytes were pre-treated (30 min) with FCCP or oligomycin before the addition of norepinephrine. Norepinephrine treatment lasted 30 min before the cells were loaded with 20 nM TMRM. (**B**) mRNA levels of *Ucp1* and (**C**) of *Atp5if1* after 1 h, 4 h, and 24 h of norepinephrine (10 μM) treatment. (**D**) ATP5IF1 protein levels following norepinephrine stimulation (10 μM for 1 h). (**E**) Comparison between ATP5A and ATP5IF1 levels in differentiated primary brown adipocytes and brown adipose tissue. (**F**) Representative blot of IF1 mutant overexpression in differentiated WT1 brown adipocytes. FCCP - Carbonyl cyanide-p-trifluoromethoxyphenylhydrazone. Statistical test: one-way ANOVA followed by LSD post hoc test. Data are expressed with individual values and mean ± SD superimposed. The exact *p*-value is displayed when $p < 0.05$.

 © The Author(s)

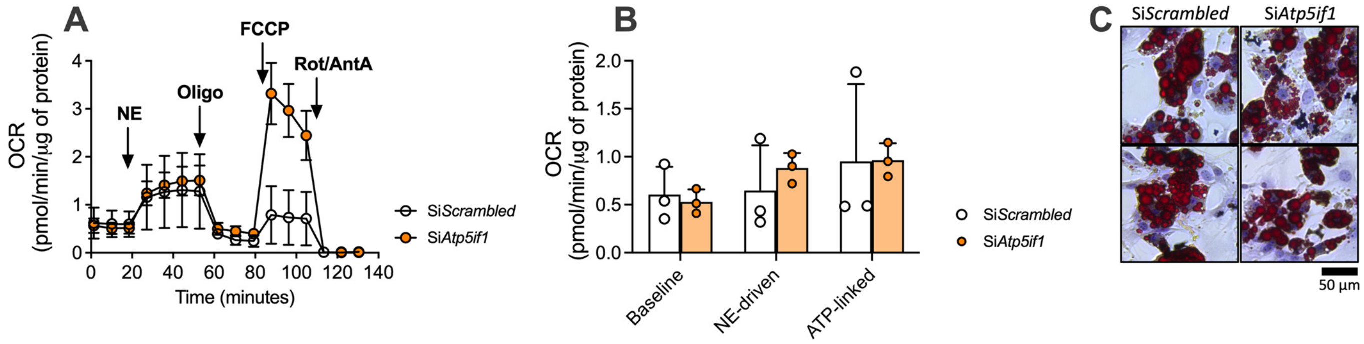

Figure EV3. Mitochondrial respiration in the presence of fatty acid-free BSA and lipid accumulation in IF1 knockdown adipocytes.

(**A**) Representative trace and (**B**) quantification of mitochondrial oxygen consumption rate in primary brown adipocytes knockdown for IF1 (si*Atp5if1*) or controls (si*Scrambled*). (**C**) Lipid content upon *Atp5if1* silencing in primary brown adipocytes visualized by Oil Red O staining. Atp5if1 ATP synthase inhibitory factor subunit 1, OCR oxygen consumption rate, NE norepinephrine, Oligo oligomycin, FCCP carbonyl cyanide-p-trifluoromethoxyphenylhydrazone, Rot rotenone, AA antimycin A. Data are expressed with individual values and mean ± SD superimposed. Two-tailed Student's t-test.

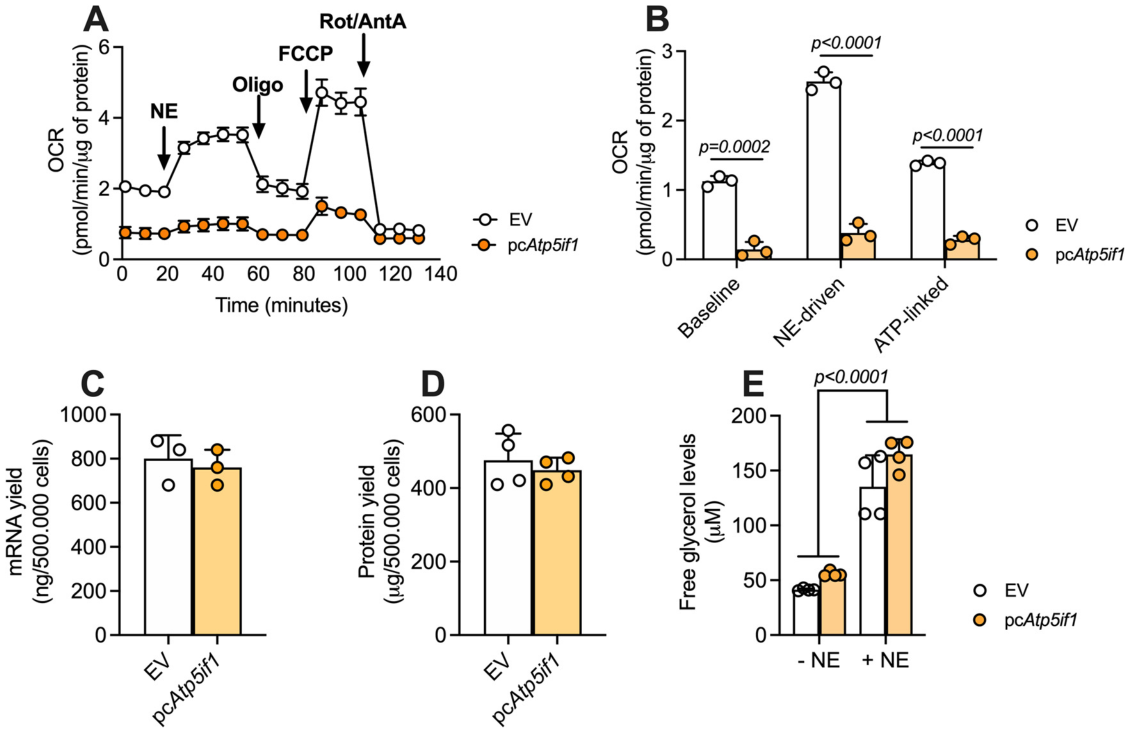

Figure EV4. IF1 overexpression suppresses mitochondrial respiration independent of free-fat acids and does not affect basic cell parameters in brown adipocytes.

(**A**) Representative trace and (**B**) quantification of mitochondrial oxygen consumption rate in primary brown adipocytes overexpressing IF1 (pcAtp5if1) or controls (EV). (**C**) mRNA and (**D**) protein yield from 500,000 cells overexpressing IF1. (**E**) Norepinephrine-induced lipolysis (10 µM for 90 min) in IF1-overexpressing adipocytes. Atp5if1 ATP synthase inhibitory factor subunit 1, OCR oxygen consumption rate, NE norepinephrine, Oligo oligomycin, FCCP carbonyl cyanide-p-trifluoromethoxyphenylhydrazone, Rot rotenone, AA antimycin A. Two-tailed Students t test (**B**, **C**, **D**). Two-way ANOVA followed by LSD post hoc test (**E**). The exact *p*-value is displayed when $p < 0.05$. Data are expressed with individual values and mean ± SD superimposed.

 © The Author(s)

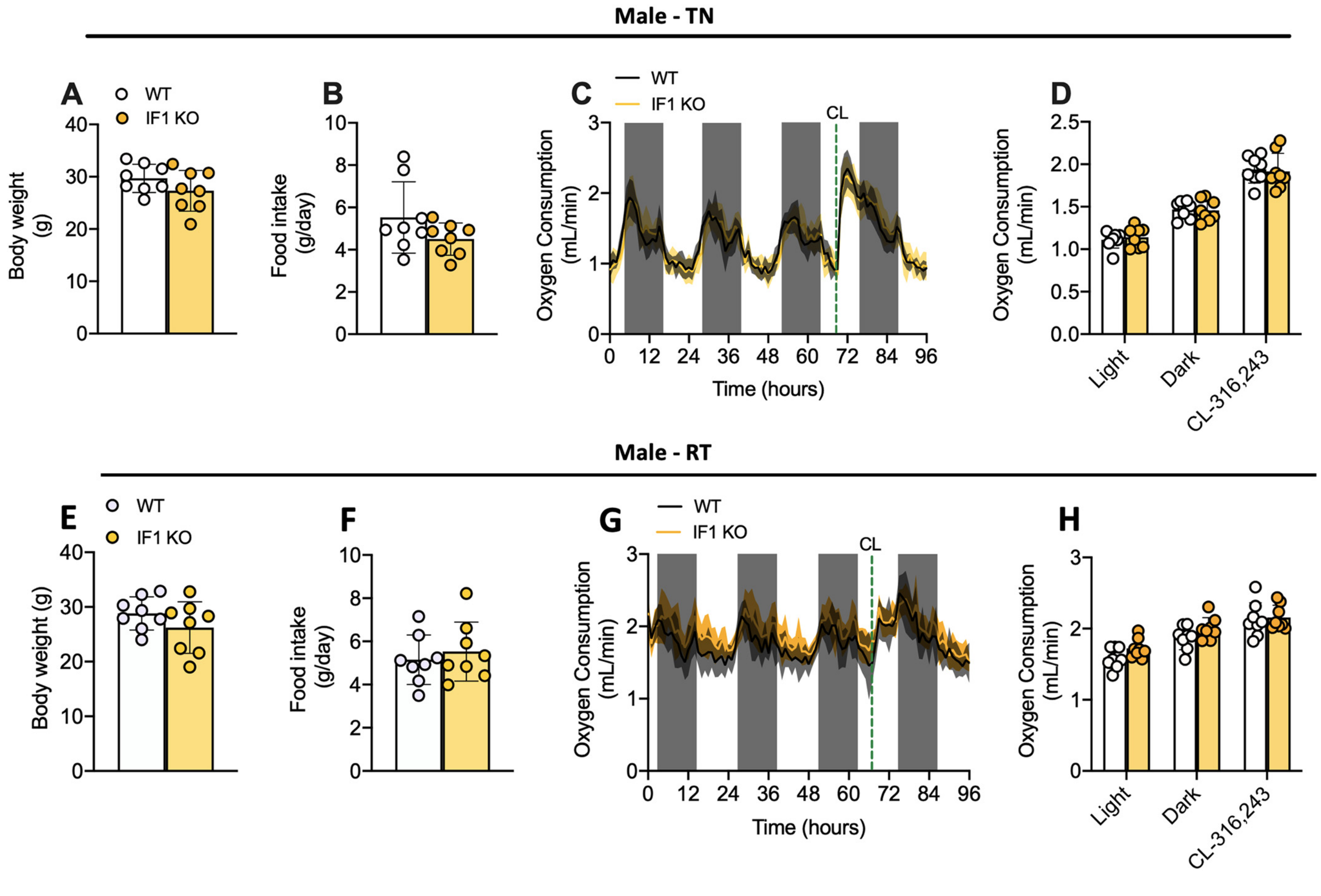

Figure EV5. IF1 global knockout does not affect resting and adrenergic-stimulated whole-body oxygen consumption.

(**A**) Body weight, (**B**) food intake, (**C**) real-time trace, and (**D**) average of baseline and CL316,243-induced energy expenditure in adult WT and IF1 KO male mice after 2 weeks of living at thermoneutrality. (**E**) Body weight, (**F**) food intake, (**G**) real-time trace, and (**H**) average of baseline and CL316,243-induced energy expenditure in adult WT and IF1 KO male mice after 2 weeks living at 23 °C. WT wild type, IF1 KO Mice with global IF1 knockout, RT room temperature, TN thermoneutrality. Two-tailed Student's t-test. Data are expressed with individual values and mean ± SD superimposed.

© The Author(s)

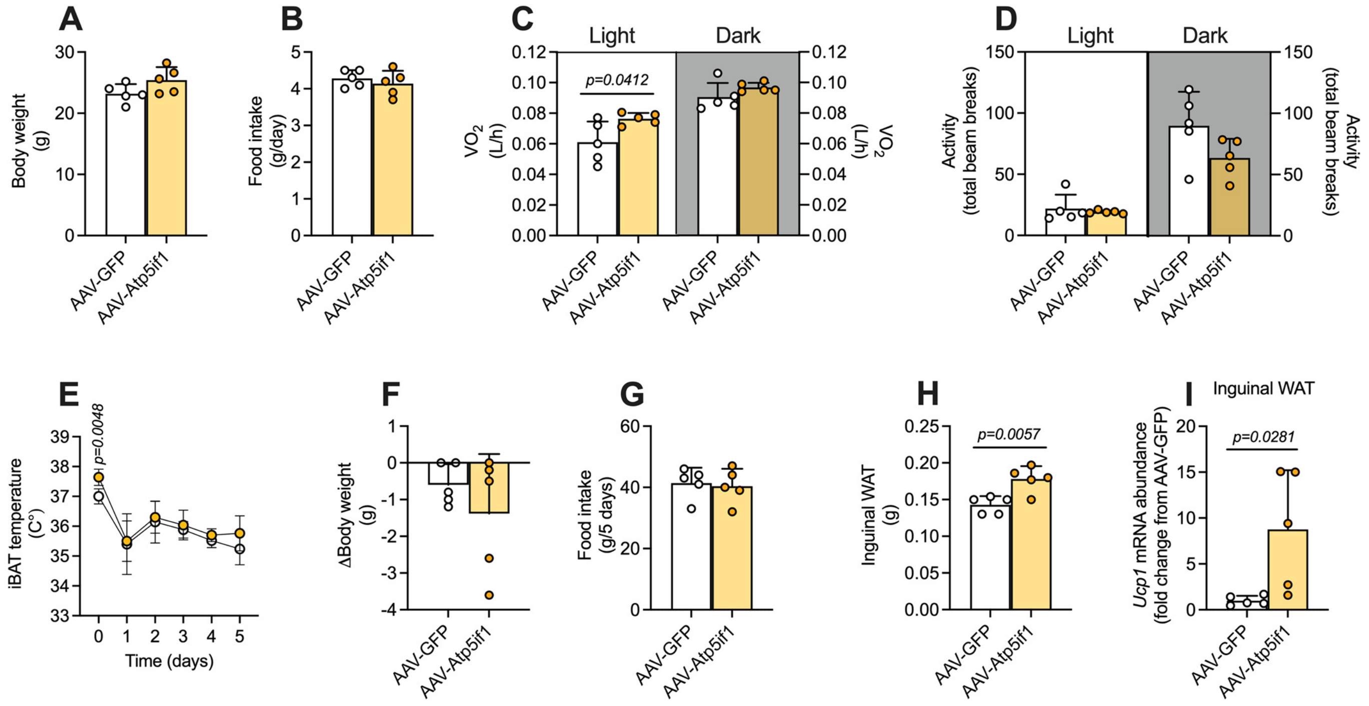

Figure EV6. Effects of BAT IF1 overexpression in male mice.

(**A**) Body weight and (**B**) daily food intake after 14 days of AAV transduction. (**C**) Oxygen consumption and (**D**) voluntary ambulatory activity in the dark and light cycles. (**E**) iBAT temperature at RT (day 0) and following cold exposure (4 °C). (**F**) Change of body weight and (**G**) food intake after 5 days of cold exposure (4 °C). (**H**) Inguinal WAT mass and (**I**) mRNA *Ucp1* levels after 5 days of cold exposure. WAT white adipose tissue. Statistical test: Two-tailed Student's t-test. The exact *p*-value is displayed when $p < 0.05$. Data are expressed with individual values and mean ± SD superimposed.

 © The Author(s)
